# Population pharmacokinetic analyses for sulbactam–durlobactam using Phase 1, 2, and 3 data

**DOI:** 10.1128/aac.00485-24

**Published:** 2024-11-21

**Authors:** Anthony P. Cammarata, M. Courtney Safir, Michael Trang, Kajal B. Larson, John P. O'Donnell, Sujata M. Bhavnani, Christopher M. Rubino

**Affiliations:** 1 Institute for Clinical Pharmacodynamics, Inc., Schenectady, New York, USA; 2 Entasis Therapeutics, Inc., an affiliate of Innoviva Specialty Therapeutics, Inc., Waltham, Massachusetts, USA; Providence Portland Medical Center, Portland, Oregon, USA

**Keywords:** sulbactam–durlobactam, sulbactam, durlobactam, population pharmacokinetics, Phase 1, Phase 2, Phase 3

## Abstract

Sulbactam–durlobactam is a β-lactam/β-lactamase inhibitor combination approved in the United States for the treatment of hospital-acquired and ventilator-associated bacterial pneumonia caused by susceptible isolates of *Acinetobacter baumannii–calcoaceticus* in adults. A population pharmacokinetic (PK) model of sulbactam–durlobactam in plasma was developed using data from eight Phase 1–3 studies. A total of 432 subjects and 8,100 plasma concentrations were available for the population PK data set. The combined model was a four-compartment (two compartments per drug) model with linear kinetics. Both renal clearance and nonrenal clearance were estimated, and total clearance was calculated as the sum of renal and nonrenal clearance. Individual renal clearances were scaled by baseline creatinine clearance. The sampling–importance–resampling analysis indicated that the parameters were estimated reliably with adequate precision. Hemodialysis (HD) and epithelial lining fluid (ELF) sub-models were developed for each analyte separately. Intermittent HD resulted in an approximately 30% decrease in the daily area under the concentration–time curve (AUC_0–24_) when HD was started 1 hour after the end of the infusion. Assuming protein binding estimates of 10% and 38% for durlobactam and sulbactam, respectively, ELF penetration ratios were found to be 41.3% for durlobactam and 86.0% for sulbactam. Of the statistically significant covariates of PK identified, which included body weight, body mass index, infection type, and region of origin, renal function was the only clinically relevant covariate. Overall, a robust description of the plasma PK of sulbactam and durlobactam was achieved. The resultant population PK model was expected to be appropriate for model-based simulations and assessment of pharmacokinetic–pharmacodynamic relationships.

## INTRODUCTION

*Acinetobacter baumannii* can cause various types of infections such as pneumonia, bacteremia, wound and soft tissue infections, urinary tract infections, and osteomyelitis (1, 2). These infections are most commonly found in intensive care unit patients (2, 3). Antimicrobial resistance in *A. baumannii*, particularly with carbapenem-resistant *A. baumannii* (CRAB), has become so prevalent and concerning that the United States Centers for Disease Control and Prevention has classified it as an “urgent threat,” and the World Health Organization has listed it as a “critical” pathogen in dire need of new antibiotics (2–4).

Durlobactam (formerly known as ETX2514) is a non-β-lactam, β-lactamase inhibitor of Class A, C, and D β-lactamase enzymes (5). Although it has no significant activity against *A. baumannii* alone, durlobactam has been shown to restore the *in vitro* and *in vivo* activity of sulbactam against multidrug-resistant *A. baumannii* when the two are used together (5–7). The efficacy of the sulbactam–durlobactam combination was also demonstrated against CRAB and other *A. baumannii–calcoaceticus* complex (ABC) isolates (8, 9). Sulbactam–durlobactam has been approved in the United States in adults for the treatment of hospital-acquired bacterial pneumonia (HABP) and ventilator-associated bacterial pneumonia (VABP) caused by susceptible isolates of ABC (10). It is not indicated for the treatment of HABP/VABP caused by pathogens other than susceptible isolates of ABC.

During the course of the development of sulbactam–durlobactam, a population pharmacokinetic (PK) model for durlobactam had been developed using data collected from a Phase 1 single ascending dose (SAD)/multiple ascending dose (MAD) study and a Phase 1 renal impairment study (11). A previously published population PK model for sulbactam was also implemented to enable model-based simulations (12). A two-compartment structural model with renal and nonrenal elimination components was found to be most appropriate for both drugs and adequately captured the observed PK data (11). Following the completion of these analyses, data from four additional Phase 1 studies (13–16), a Phase 2 study in patients with complicated urinary tract infections (cUTI) (17), and a Phase 3 study in patients with infections caused by ABC (18) became available. The objective of these analyses was to pool these additional Phase 1, 2, and 3 data with the Phase 1 data evaluated in prior analyses to develop a combined population PK model for sulbactam–durlobactam.

## RESULTS

### Data

A total of 432 subjects and 8,100 plasma concentration records, including 5,275 durlobactam concentrations from 432 subjects and 2,825 sulbactam concentrations from 303 subjects, were available from the eight studies. Each study is described in Table S1. Details of the available number of plasma and epithelial lining fluid (ELF) samples and subjects by study and reasons for exclusion of samples and subjects/patients are provided in Table S2. Samples below the limit of quantitation (BLQ) drawn after a sulbactam–durlobactam dose comprised approximately 14% of the samples in the final data set (905 out of 6,343 samples). While this number was relatively high, it resulted from the short half-life of the drugs and the extended sampling scheme employed in the Phase 1 studies; less than 1% of BLQ samples occurred in the first 12 hours after a dose, while 30% were between 24 and 36 hours after a dose, and 69% occurred ≥48 hours after a dose. Overall, only 48 outliers were identified and excluded out of a total of 5,438 available plasma concentration records (<1%). After the removal of excluded records and outliers, the population PK model development data set contained 373 subjects and 5,390 plasma concentration records. The final population PK model development data set for plasma contained 373 subjects and 5,188 concentration records; 202 concentration records from Period 2 were for intermittent hemodialysis (HD) subjects enrolled in Cohort 5 of Study CS2514-2017-0002 and were not part of the final plasma PK model but rather the HD sub-model. When stratified by analyte, a total of 373 subjects provided 3,494 durlobactam concentrations and 264 subjects provided 1,944 sulbactam concentrations to the final data set. A total of 60 ELF concentration records were available from 30 subjects from Study CS2514-2017-0001 for the construction of the ELF sub-model.

Summary statistics of baseline subject descriptors for the analysis population are presented in Table S3. In general, body size was similar across studies with the exception of the Chinese subjects enrolled in Study ZL-2402-001, who had smaller body size metrics [height, weight, body surface area (BSA), and body mass index (BMI)]. Patients enrolled in the renal impairment study (Study CS2514-2017-0002), Phase 2, and Phase 3 studies were slightly older than those enrolled in the other Phase 1 studies. Renal function, as measured by creatinine clearance (CLcr), and which was calculated using the Cockcroft–Gault equation (19) and normalized to the body surface area, was relatively consistent across studies with the exception of Study CS2514-2017-0002. The lower median CLcr in Study CS2514-2017-0002 was likely a result of the inclusion of patients with CLcr as low as approximately 6 mL/min/1.73 m^2^. Overall, the population was slightly biased toward male subjects (62.5%); 75% of the subjects in the Phase 3 study were male. Of note, the pooled population was predominantly white (65.4% overall).

**Fig 1:**
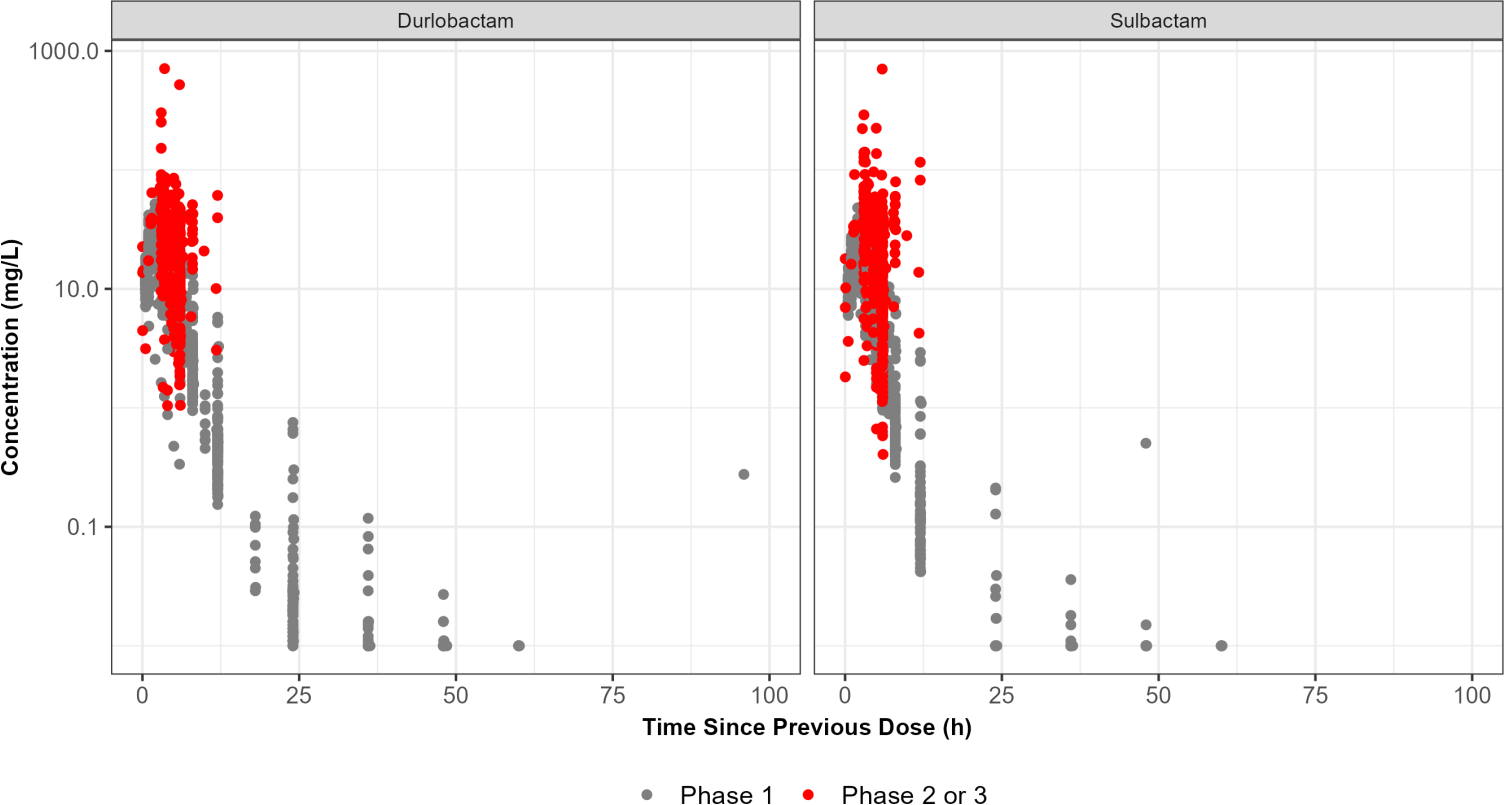
Semi-log plot of plasma concentrations versus time since previous dose, stratified by phase and paneled by analyte. Note: h, hours; L, liters; mg, milligrams.

Plots of plasma concentrations versus time since previous dose, stratified by phase and paneled by analyte, are presented in Fig. 1. When the Phase 2 and 3 data are overlaid atop the Phase 1 data, it was evident that, while the concentrations were slightly higher in patients, the data from Phases 2 and 3 remained within the range of the Phase 1 data. Similar results were seen across both durlobactam and sulbactam. Plots of plasma concentrations versus time since previous dose, stratified by infection type and paneled by analyte, are presented in Fig. S1. In Fig. S1, there tended to be more observed concentrations from patients above the 95th confidence interval from Phase 1 studies across the PK sampling interval. Despite the variability seen in the durlobactam and sulbactam concentrations in patients, distinct trends by infection type were not apparent.

Plots of plasma concentrations versus time, stratified by analyte and paneled by phase and study, are presented in Fig. S2. Overall, the PK profiles were consistent with intensive PK sampling schemes utilized for single- and multi-dose cohorts in Phase 1 studies and sparse sampling for Phase 2 and 3 studies. Variation in dosing appeared to be linear with higher peaks observed in higher dosing regimens.

Plots of mean plasma concentrations versus time since previous dose, stratified by study (Chinese Phase 1 study vs other Phase 1 studies where subjects received 1 g sulbactam/1 g durlobactam) and paneled by analyte, are presented in Fig. S3. Since all data shown in this figure were from Phase 1 studies with intensive sampling schemes with 1 g sulbactam/1 g durlobactam dosing, the full PK curves could be clearly seen. Overall, the concentrations over time for durlobactam in subjects enrolled in the Chinese Phase 1 study were higher than those from the other Phase 1 studies. However, concentrations over time for sulbactam showed little difference between the Chinese Phase 1 study and all other Phase 1 studies.

### Population pharmacokinetic model development

The previous population PK model was fit to the pooled Phase 1, 2, and 3 data, with CLcr (normalized to BSA in order to avoid confounding between body size and renal function) included in the base structural models *a priori*. The fraction of durlobactam and sulbactam assumed to be excreted renally (FE) was fixed to the values that had been used during the previous population PK analyses conducted for sulbactam–durlobactam (0.479 for both drugs) (11). However, during a comprehensive review of urine data collected across the clinical development program for sulbactam–durlobactam, variability in the assessment of FE was noted, which could be due to the handling and processing of human urine samples. Therefore, it was decided to use the FE from subjects with normal renal function from Study CS2514-2017-0002 as a more reliable estimate of FE. This change was made after the completion of the combined population PK model for both durlobactam and sulbactam. The final model was, therefore, refit using the more reliable estimates for FE. Fitting the model to the pooled Phase 1, 2, and 3 data provided an adequate fit to the data based upon traditional goodness-of-fit plots, and the model parameters appeared to be estimated robustly. However, after further evaluation, some bias in the overall fit was observed. Therefore, attempts were made to improve the model by evaluating the structural model components. In addition, further exploration of potential outliers was performed. Ultimately, a relatively small number of outliers from Phase 3 subjects were excluded (*n* = 48), and an adequate fit of the base models was obtained (data not shown).

During base model development, time-varying covariates were explored and, if deemed necessary, tested to determine if they were relevant to the model. The use of time-varying CLcr was compared against the use of baseline CLcr in the models for renal and nonrenal clearances for both analytes. Baseline CLcr was found to be more appropriate (i.e., there was no improvement in the minimum value of the objective function when using time-varying CLcr) and was, therefore, used throughout the modeling process.

Of note, the base model development was carried out separately for both durlobactam and sulbactam even though the base models were very similar for each. The base structural population PK models for durlobactam and sulbactam in plasma, which best described the pooled data from Phase 1, 2, and 3 studies, each contained two compartments (central and peripheral) with linear, first-order elimination. Consistent with previous analyses, relationships between clearance (CL) and CLcr were included in the base structural models *a priori*, which indicated that CLcr was a significant predictor of the interindividual variability (IIV) in the CL of both drugs (11).

The base structural models described above were utilized for the subsequent covariate analysis. The potential impact on PK of all physiologically relevant covariates was explored statistically for both durlobactam and sulbactam. Relationships between renal and nonrenal clearance were not explored further as they had already been incorporated as part of the base structural model development process.

All of the covariate relationships identified during forward selection were retained after backward elimination as the removal of any of the relationships resulted in significant increases in the objective function. Evaluation of the resultant model indicated that changes were necessary for each respective model. For the durlobactam model, the separate terms describing the relationships between volume of distribution in the central compartment (Vc) and infection type for HABP and VABP were combined as they did not yield significantly different effects on PK from one another. Additionally, the additive component of residual variability (RV) on Phase 3 data was determined to not be significant and was consequently removed. For both the durlobactam and sulbactam models, patients with CLcr <30 mL/min/1.73 m^2^ were found to produce significantly different results from other patients with higher CLcr values. Total CL was, therefore, adjusted for patients with CLcr <30 mL/min/1.73 m^2^ using a proportional shift. All parameter–covariate relationships were described with a proportional shift with the exception of CLcr and weight, which were described using power functions.

Following backward elimination, the separate models for durlobactam and sulbactam were combined to construct the final population PK model for plasma. The combined model was a four-compartment (two compartments per drug) model with linear kinetics. Both renal clearance and nonrenal clearance were estimated, and total CL was calculated as the sum of renal and nonrenal clearance. Individual renal clearances were scaled by baseline CLcr, and the total CL for subjects with CLcr below 30 mL/min/1.73 m^2^ was adjusted to account for the apparent differences in that sub-population. Interindividual variability was estimated for CL, Vc, and volume of distribution in the peripheral compartment (Vp) with off-diagonal relationships on CL and Vc for both sulbactam and durlobactam. Residual variability was described for durlobactam as a series of proportional and additive error models for plasma concentrations obtained from subjects enrolled in Phase 1, 2, and 3 studies, respectively. For sulbactam, RV was described by one proportional and additive model for all of the data.

The final population PK parameter estimates and associated standard errors for the model are provided in Table 1 along with the resample statistics from the sampling–importance–resampling (SIR) analysis. The resampled parameter means were aligned with those estimated in the final model fit, with 95% confidence intervals consistent with the precision of the final model. This indicated that the parameters were estimated reliably with adequate precision.

**TABLE 1:** Summary statistics of resampled population PK parameters in comparison to the model parameter estimates from the final population pharmacokinetic model^*a*^

Parameter	Final model			Resample statistics			
	Estimate	%SEM	Shrink.	Mean	Median	%CV	90% CI
Durlobactam							
CL (L/h)	9.33	3.24		9.34	9.33	1.86	[9.07, 9.65]
CL_R, CLcr power_	0.875	12		0.877	0.875	7.54	[0.766, 0.988]
Vc (L)	12.5	2.93		12.5	12.5	2.08	[12.1, 12.9]
*Q* (L/h)	4.43	3.77		4.43	4.43	3.82	[4.17, 4.71]
Vp (L)	5.83	3.44		5.82	5.83	2.83	[5.54, 6.10]
FE (%)	0.479						
CLEASIAFL1	−0.199	31.7		−0.198	−0.197	24.9	[−0.282, −0.117]
CLWTKG1	0.646	19.4		0.651	0.648	12.1	[0.527, 0.783]
V1INFTYPN1&2 (HABP and VABP)	1.52	26.5		1.54	1.53	17.4	[1.12, 2.01]
V1INFTYPN3 (cUTI)	0.343	62.3		0.348	0.344	32.4	[0.165, 0.532]
V1INFTYPN4 (bacteremia)	3.32	53.7		3.31	3.33	22.6	[2.05, 4.52]
V1WTKG1	0.521	27.0		0.524	0.521	20.3	[0.359, 0.704]
V1EASIAFL1	−0.263	49.2		−0.257	−0.257	25.8	[−0.369, −0.147]
BCLCRNLT30	−0.58	8.15		−0.581	−0.58	3.62	[−0.617, −0.547]
ω^2^_CL_	0.0778 (27.9 %CV)	9.56	9.13	0.0781	0.0778	7.67	[0.0684, 0.0882]
ω^2^_Vc_	0.0757 (27.5 %CV)	13.6	25.7	0.0759	0.0759	10.8	[0.0626, 0.0895]
ω^2^_Vp_	0.0773 (27.8 %CV)	9.83	37.6	0.0777	0.0771	9.7	[0.0668, 0.0909]
Covariance (ω^2^_CL_, ω^2^_Vc_)	0.0494 (*r*^2^ = 0.415)	17.8		0.0494	0.0493	13.9	[0.0377, 0.0608]
σ^2^_plasma, Proportional Phase 1_	0.019 (13.8 %CV)	1.90	8.67	0.019	0.019	2.01	[0.0183, 0.0196]
σ^2^_plasma, Additive Phase 1_	0.00136 (0.0369 mg/L)	3.69	8.67	0.00136	0.00136	4.91	[0.00125, 0.00147]
σ^2^_plasma, Proportional Phase 2_	0.0794 (28.2 %CV)	13.3	12.1	0.0795	0.0792	9.86	[0.0678, 0.0928]
σ^2^_plasma, Proportional Phase 3_	0.203 (45.0 %CV)	9.85	8.61	0.203	0.202	9.69	[0.173, 0.238]
Sulbactam							
CL (L/h)	13.5	14.0		13.4	13.3	5.2	[12.3, 14.6]
CL_R, CLcr power_	1.14	20.8		1.14	1.14	11.7	[0.920, 1.37]
Vc (L)	12	8.90		12.0	12.0	5.32	[10.9, 13.0]
*Q* (L/h)	7.88	18.9		8.01	7.92	12.4	[6.56, 9.72]
Vp (L)	6.99	9.29		7.01	7.01	6.74	[6.24, 7.80]
FE (%)	0.479						
CLINFTYPN1 (HABP)	−0.424	19.4		−0.419	−0.421	10.8	[−0.494, −0.346]
CLINFTYPN2 (VABP)	−0.298	36.5		−0.294	−0.295	19.8	[−0.393, −0.195]
CLINFTYPN3 (cUTI)	−0.157	96.8		−0.146	−0.149	52.3	[−0.274, −0.00759]
CLINFTYPN4 (bacteremia)	−0.444	20.9		−0.437	−0.438	13.9	[−0.537, −0.333]
CLINFTYPN5 (AP)	−0.382	28.8		−0.373	−0.374	18.2	[−0.489, −0.253]
CLWTKG1	1.01	26.9		1.01	1.01	13.7	[0.795, 1.24]
V3INFTYPN1 (HABP)	0.836	25.3		0.85	0.846	21.6	[0.554, 1.15]
V3INFTYPN2 (VABP)	1.43	19.5		1.44	1.44	17.1	[1.04, 1.84]
V3INFTYPN3 (cUTI)	0.17	89.8		0.179	0.177	65.6	[−0.00793, 0.362]
V3INFTYPN4 (bacteremia)	1.85	44.7		1.9	1.88	34.4	[0.911, 2.96]
V3INFTYPN5 (AP)	−0.704	13.2		−0.703	−0.703	11	[−0.824, −0.579]
V3WTKG1	0.831	26.7		0.836	0.829	18.9	[0.573, 1.11]
BCLCRNLT30	−0.635	11		−0.632	−0.633	5.07	[−0.682, −0.578]
ω^2^_CL_	0.221 (47.0 %CV)	10.6	19.2	0.223	0.223	7.99	[0.194, 0.252]
ω^2^_Vc_	0.0967 (31.1 %CV)	24.5	44.7	0.097	0.0963	20.1	[0.0667, 0.131]
ω^2^_Vp_	0.196 (44.3 %CV)	25.7	50.0	0.199	0.197	19.6	[0.141, 0.267]
Covariance (ω^2^_CL_, ω^2^_Vc_)	0.0727 (*r*^2^ = 0.2448)	32.6		0.0725	0.072	25.8	[0.0428, 0.104]
σ^2^_plasma, Proportional_	0.0433 (20.8 %CV)	2.54	11.6	0.0433	0.0433	2.73	[0.0413, 0.0451]
σ^2^_plasma, Additive_	0.0054 (0.0735 mg/L)	6.38	11.6	0.00537	0.00538	6.81	[0.00478, 0.00596]

^*a*^ Shaded regions are values that were fixed or not estimated. AP, acute pyelonephritis; CI, confidence interval; CL_R_, renal clearance; %CV, percent coefficient of variation; *n*, number of observations or subjects; *Q*, distributional clearance; %SEM, percent standard error of the mean; ω^2^, interindividual variability; σ^2^, residual variability; WTKG, body weight in kilograms.

The primary goodness-of-fit plots for the final population PK model are provided in Fig. S4. These plots demonstrated the adequacy of the model fit across healthy subjects and infected patients. The normalized prediction distribution error (NPDE) plots shown in Fig. S5 indicated little to no bias in the fit across the range of data.

**Fig 2:**
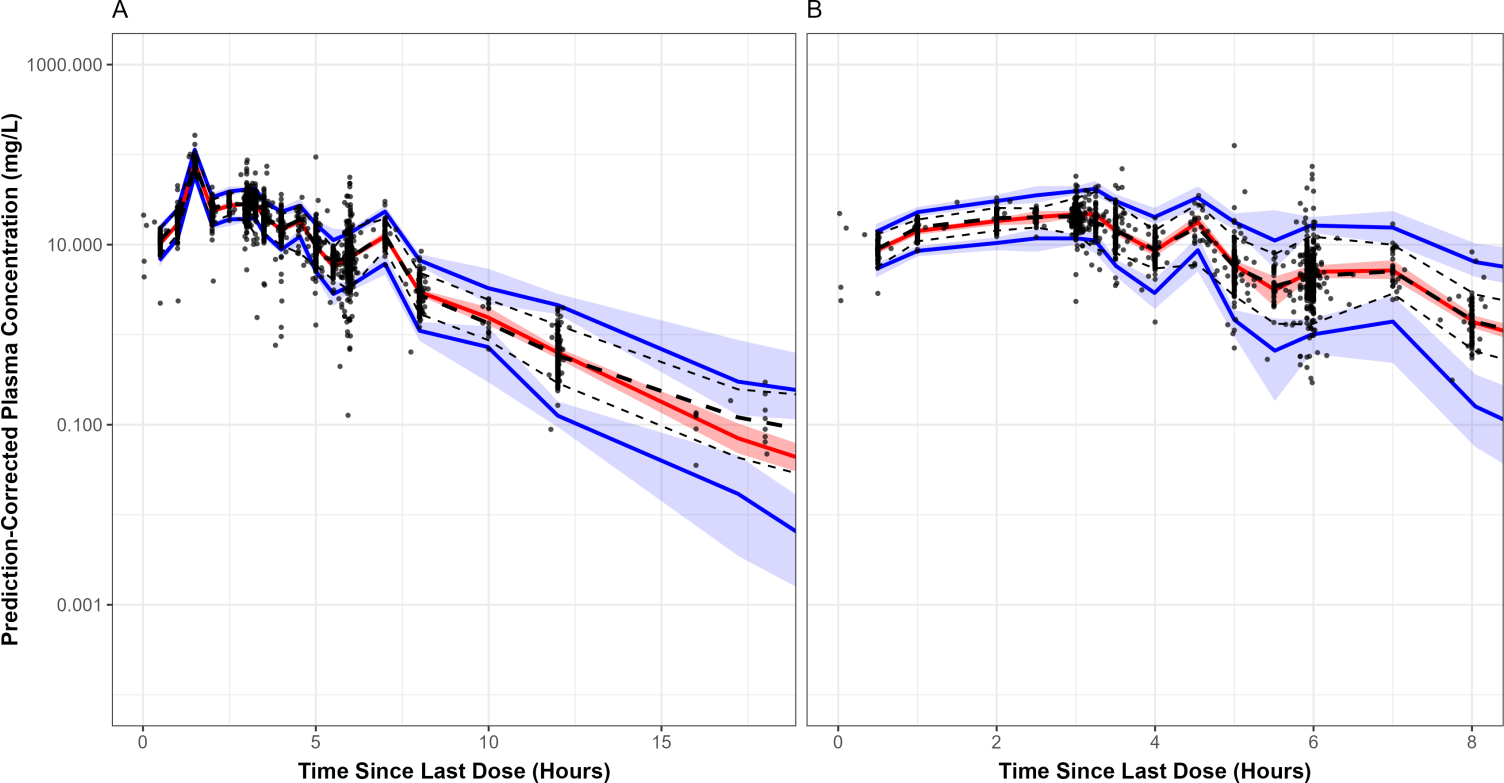
Prediction-corrected visual predictive check plot for the final model of durlobactam (**A**) and sulbactam (**B**) using the pooled analysis data set, pooled by analyte. Circles are observed concentrations, black long dashed lines are the median observed concentrations, and black short dashed lines are the 5th and 95th percentiles of the observed concentrations. Red and blue shaded regions are the 90% confidence intervals for the median, 5th, and 95th percentiles from the simulations; the red and blue lines represent the medians of these regions.

Prediction-corrected visual predictive check (PC-VPC) plots showed good agreement between median simulated plasma concentrations based on the final population PK model and the median observed plasma concentrations for both the durlobactam and sulbactam models (Fig. 2) and when only healthy subjects or patients are included (Fig. S6 and S7, respectively). Overall, the model was less robust in capturing the variability in the observed concentrations as the 5th and 95th percentiles of the observed data did not universally fall within the confidence intervals of the corresponding simulated values. However, the degree of bias was small and acceptable given the observed within-subject variability in drug concentrations over time and the intended purpose of this model.

Overall, this model was expected to provide robust and reliable estimates of sulbactam–durlobactam plasma exposures in Phase 3 patients for use in pharmacokinetic–pharmacodynamic (PK-PD) analyses for efficacy (20). The simulation-based diagnostics also suggested that the model was appropriate for the conduct of PK-PD target attainment analyses (20, 21).

As previously mentioned, the original estimates for FE for the two compounds were revised. To assess the impact of altering FE on the fit of the population PK model, the final dual-drug model was refit using the corrected FE values of 0.660 for durlobactam and 0.648 for sulbactam, derived from the subjects with normal renal function enrolled in Study CS2514-2017-0002. Examination of these parameters (data not shown) relative to those obtained for the original dual-drug model revealed that the only parameters that were altered to a noticeable extent were those that are associated with the relationships between CL and CLcr for either drug. Overall, the IIV in the PK parameters either remained consistent (for the volume parameters) or decreased slightly. The parameters defining the residual variability model were altered by less than 2%. Overall, refitting the model with the updated FE estimates did not alter the fit appreciably. The goodness-of-fit plots still demonstrated the adequacy of the model fit across healthy subjects and patients, and the PC-VPC plots showed good agreement between median simulated plasma concentrations based on the refitted population PK model and the median observed plasma concentrations for both the durlobactam and sulbactam models, regardless of the study population. Even though the estimation of the FE terms did not alter the model parameters and overall model fit significantly, the refitted, dual-drug model was used for the calculation of individual, *post hoc* exposure estimates in the PK population and was also used as the basis for PK-PD target attainment simulations (20, 21).

### Hemodialysis sub-models

Hemodialysis sub-models were developed for each analyte separately. The final HD sub-models were nearly the same as the final plasma population PK model. However, a few key differences were present that allowed for distinction between the models. The plasma terms were all fixed to the population mean parameter values with IIV applied for each subject, and an HD effect (HDEFFECT) term with IIV was applied to CL for each of the two analyte sub-models. HDEFFECT is the estimation of a theta term with an ETA term that affects CL only. The CL-HDEFFECT parameters were simply multiplicative terms with IIV applied to them so they varied by subject. This meant that the effects of HD with durlobactam and sulbactam caused an increase in CL by 6.24-fold and 8.19-fold, respectively.

The PK parameter estimates and associated standard errors for the HD sub-models for both durlobactam and sulbactam are provided in Table S4. To illustrate the impact of intermittent HD on the daily area under the concentration–time curve (AUC_0–24_), a simulation was conducted using the individual *post hoc* PK parameter estimates for each of the subjects in the HD cohort. Summary statistics for the key durlobactam and sulbactam PK exposure parameters are provided in Table S5 for subjects enrolled in the HD cohort of the renal impairment study. Results demonstrated that administering intermittent HD resulted in an approximately 30% decrease in AUC_0–24_ when HD was started 1 hour after the end of the infusion for the morning dose and continued for 4 hours. This represents a “worst-case” scenario as the impact on AUC_0–24_ would be less if dialysis was started later in the dosing interval (e.g., if the dose was given after the completion of HD). The area under the concentration–time curve from time 0 to 8 hours (AUC_0–8_) and AUC from time 0 to 12 hours (AUC_0–12_) estimated for Period 2 (when the drug was administered before the start of HD), while lower than those observed in Period 1 (when the dose was administered after an HD session), did not fall by the same extent as the predicted increase in CL described above. The maximum plasma concentration (*C*_max_) was observed before HD began in Period 2 and was, therefore, not impacted by the effect of HD.

Goodness-of-fit plots (Fig. S8 and S9) for the HD sub-models demonstrated the adequacy of the model fit across patients undergoing HD from Study CS2514-2017-0002. The NPDE plots (data not shown) indicated little to no bias in the fit across the range of data. Any bias seen in the plots was likely due to the relatively limited number of subjects and time points where HD occurred.

### ELF sub-models

Similar to the hemodialysis sub-models, the ELF sub-models for each analyte were developed separately and were nearly identical to the final plasma population PK model. In the ELF sub-models, the plasma terms are all fixed to the population mean parameter values with IIV applied for each subject, and a plasma to ELF ratio term was estimated for each of the two analyte sub-models. Employment of a ratio term meant no specific parameters were affected.

The PK parameter estimates and associated standard errors for the ELF sub-models for both durlobactam and sulbactam are provided in Table S6. Given that the ELF sub-models assumed that ELF concentrations were simply a ratio of the plasma concentrations (i.e., with no time lag), the population mean estimates indicated that the ELF penetration relative to total-drug plasma concentrations for durlobactam was 37.2%, and the ELF penetration for sulbactam was 53.3%. Assuming protein binding estimates of 10% and 38% for durlobactam and sulbactam, respectively (13, 22), the model-derived ELF penetration ratios relative to free-drug plasma concentrations were 41.3% and 86.0% for durlobactam and sulbactam, respectively. Summary statistics for the key durlobactam and sulbactam AUC_0–24_ and *C*_max_ in plasma and ELF are provided in Table S7 for subjects enrolled in the ELF penetration study (Study CS2514-2017-0001).

Goodness-of-fit plots (Fig. S10 and S11) for the ELF sub-models demonstrate the adequacy of the model fit across patients that were sampled for ELF from Study CS2514-2017-0001. The NPDE plots (data not shown) indicated little to no bias in the fit across the range of data. Any bias seen in the plots was likely due to the limited number of subjects and time points where ELF was collected.

### Comparison of key sub-groups

**Fig 3:**
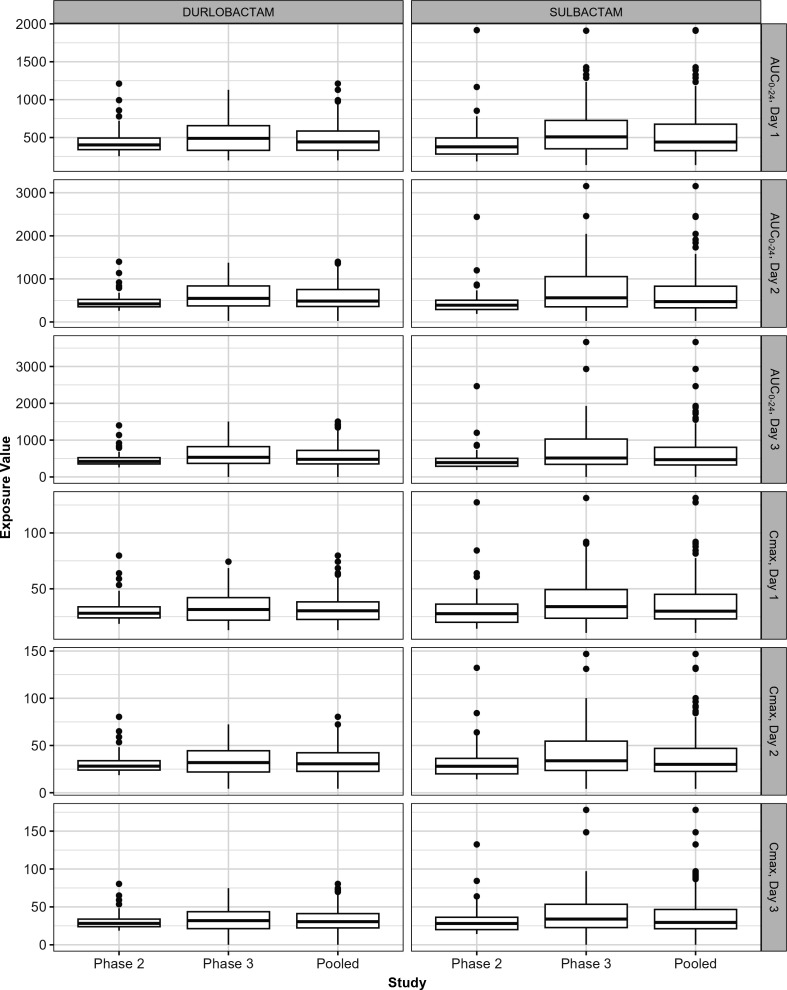
Box-and-whisker plots showing the distributions of AUC_0–24_ and *C*_max_ by day and study phase. Counts of observations per group are provided in Table 2.

Summary statistics for the key durlobactam and sulbactam PK exposure parameters (AUC_0–24_ and *C*_max_ on Days 1 through 3) and CL, Vc, volume of distribution at steady state (Vss), alpha half-life (*t*_1/2,α_), and beta half-life (*t*_1/2,β_) are provided in Table 2 for subjects in Phase 2 and 3 studies, stratified by drug and study phase. Box-and-whisker plots showing the distributions of AUC_0–24_ and *C*_max_ on Days 1 through 3 by drug and study phase are provided in Fig. 3. Although exposures were slightly higher in subjects enrolled in the Phase 3 study, there was substantial overlap in the distributions suggesting that the impact of infection type [cUTI including acute pyelonephritis (AP) in Phase 2 and HABP/VABP/bacteremia due to ABC in Phase 3] on PK was not clinically relevant.

**TABLE 2:** Summary statistics [geometric mean (%CV)] for key exposures and PK parameters for subjects enrolled in Phase 2 and 3 studies, stratified by drug and study phase

Parameter	Durlobactam			Sulbactam		
	Phase 2(*n* = 52)	Phase 3(*n* = 110)	Pooled(*n* = 162)	Phase 2(*n* = 52)	Phase 3(*n* = 110)	Pooled(*n* = 162)
AUC_0–24_, Day 1 (mg•h/L)	423 (33.2%)	473 (40.3%)	456 (38.4%)	396 (46.1%)	504 (56.3%)	466 (54.3%)
AUC_0–24_, Day 2 (mg•h/L)^*a*^	448 (35.6%)	533 (62.1%)	504 (55.4%)	410 (48.1%)	577 (74.0%)	517 (68.5%)
AUC_0–24_, Day 3 (mg•h/L)	448 (35.6%)	453 (122%)	451 (103%)	411 (48.2%)	504 (117%)	472 (100%)
*C*_max_, Day 1 (mg/L)	29.4 (30.9%)	31.0 (40.1%)	30.5 (37.4%)	28.6 (44.8%)	33.7 (53.3%)	32.0 (51.1%)
*C*_max_, Day 2 (mg/L)^*a*^	29.5 (31.1%)	31.4 (49.5%)	30.8 (44.5%)	28.7 (45.2%)	34.5 (60.0%)	32.5 (56.2%)
*C*_max_, Day 3 (mg/L)	29.5 (31.1%)	27.8 (101%)	28.3 (85.1%)	28.7 (45.2%)	31.4 (94.4%)	30.5 (81.8%)
CL (L/h)	8.93 (35.6%)	7.52 (60.4%)	7.95 (54.2%)	9.74 (48.2%)	6.96 (81.5%)	7.75 (74.1%)
Vc (L)	15.7 (32.8%)	30.2 (42.3%)	24.4 (50.0%)	9.43 (78.7%)	24.4 (38.7%)	18.0 (70.4%)
Vss (L)	21.6 (23.8%)	36.4 (35.9%)	30.8 (40.5%)	16.6 (54.1%)	31.6 (31.4%)	25.7 (50.1%)
*t*_1/2,α_ (h)	0.533 (15.7%)	0.702 (11.6%)	0.643 (18.3%)	0.243 (55.8%)	0.437 (17.8%)	0.362 (44.2%)
*t*_1/2,β_ (h)	2.06 (22.0%)	3.59 (51.3%)	3.01 (51.1%)	1.51 (40.2%)	3.36 (70.4%)	2.60 (72.6%)

^*a*^ Note that exposures on Day 2 are often higher than those from Day 3 in the Phase 3 study due to the imbalance in the number of doses received on Day 2 (i.e., many subjects received an “extra” dose on Day 2 in the Phase 3 study because they were shifted to standard hospital dosing times).

Summary statistics for the key PK exposure parameters (AUC_0–24_ and *C*_max_ on Days 1 through 3) and CL, Vc, Vss, *t*_1/2,α_, and *t*_1/2,β_ stratified by CLcr group (in mL/min), body weight category, BMI category, sex, infection type, and region of origin are provided in Tables S8 to S13, respectively. Box-and-whisker plots showing the distributions of AUC_0–24_ and *C*_max_ on Days 1 through 3 by drug and renal function, body weight, BMI, age, infection type, and region are provided in Fig. S12 to S17, respectively.

A statistically significant relationship was identified between CL and renal function (as approximated by CLcr in mL/min/1.73 m^2^) for both drugs whereby drug clearance increases with increasing CLcr. Subjects with severe renal impairment (CLcr <30 mL/min) had substantially higher geometric mean exposures for both drugs than subjects with CLcr ≥60 mL/min despite the fact that the study protocol stipulated that subjects with CLcr <30 mL/min were to receive sulbactam–durlobactam using extended dosing intervals of 8–12 hours. Body weight was identified as a statistically significant predictor of the variability in CL and Vc for durlobactam and sulbactam; although there was a trend for AUC_0–24_ to increase with both decreasing body weight and BMI, BMI was not identified as statistically significant. Infection type was identified as a statistically significant predictor of the variability in Vc for durlobactam and CL and Vc for sulbactam. Neither race nor country of origin was identified as a statistically significant predictor of the variability in the PK of either durlobactam or sulbactam when evaluated as part of the formal covariate analysis. However, the East Asian region was identified as a statistically significant predictor of the variability in CL and Vc for durlobactam (but not sulbactam).

**Fig 4:**
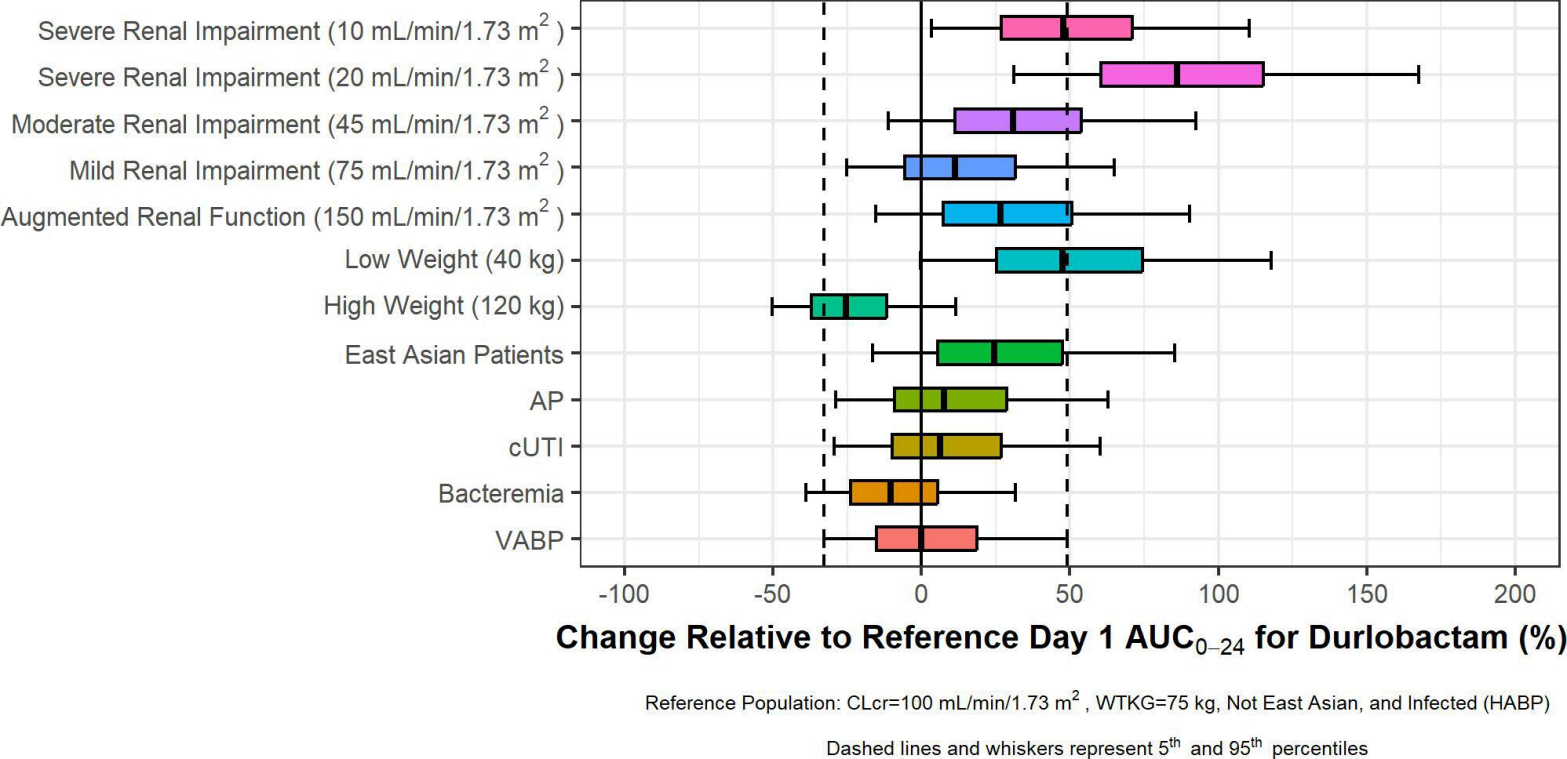
Forest plots showing impact of statistically significant covariate–parameter relationships on durlobactam AUC_0–24_ on Day 1. Note: CLcr, creatinine clearance. The predicted durlobactam exposures are shown as horizontal boxplots showing the median, interquartile range, and 5th–95th percentile of the predicted exposures. These distributions are then compared to the median and 5th–95th percentile for the base case described above. Subjects with renal impairment have been given the dose stipulated in the Phase 3 protocol for subjects with renal impairment.

**Fig 5:**
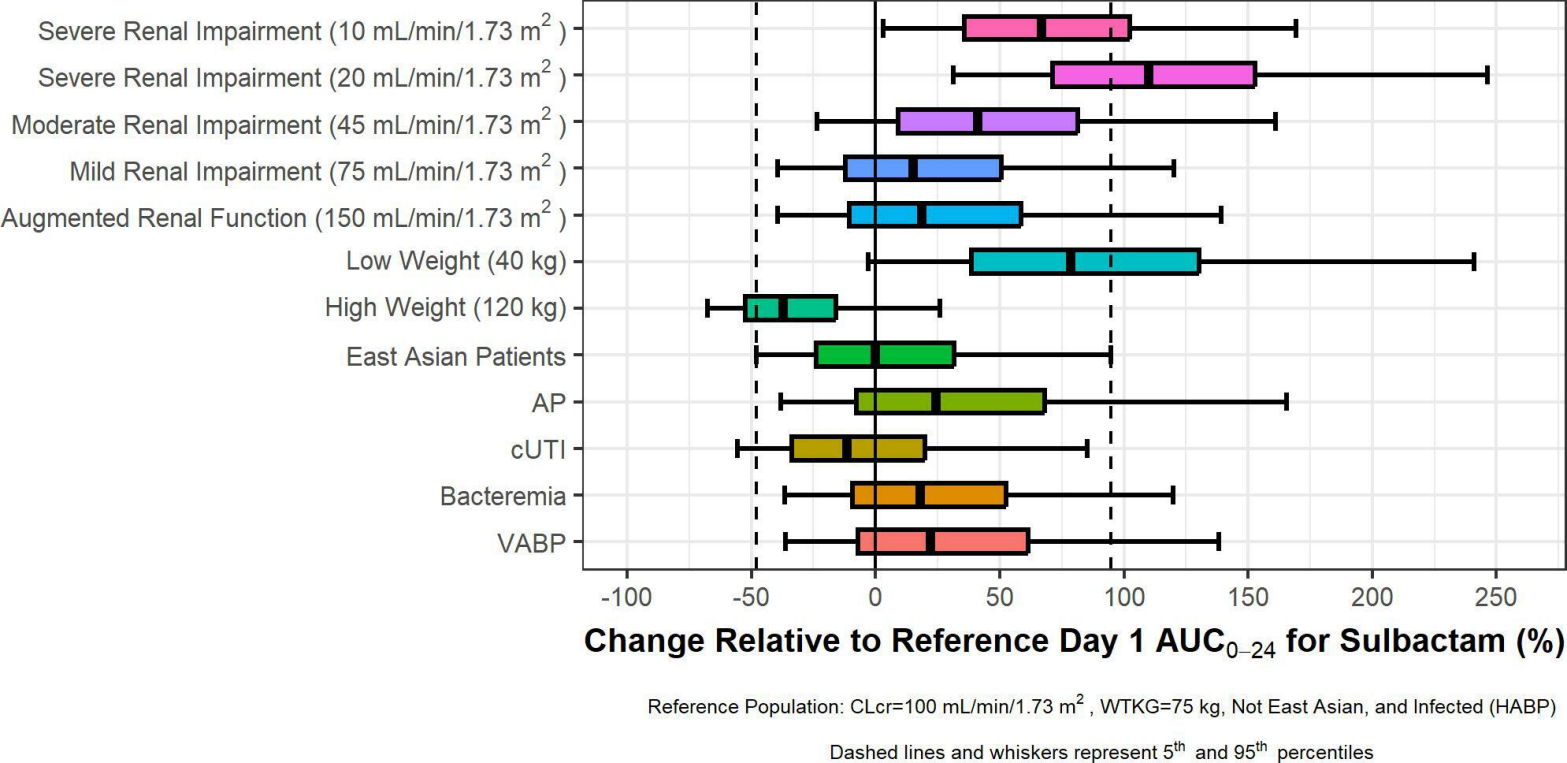
Forest plots showing impact of statistically significant covariate–parameter relationships on sulbactam AUC_0–24_ on Day 1. The predicted sulbactam exposures are shown as horizontal boxplots showing the median, interquartile range, and 5th–95th percentile of the predicted exposures. These distributions are then compared to the median and 5th–95th percentile for the base case described above. Subjects with renal impairment have been given the dose stipulated in the Phase 3 protocol for subjects with renal impairment.

The potential clinical significance of the statistically significant covariates was assessed by evaluating the predicted durlobactam and sulbactam exposures in various sub-groups. Simulated AUC_0–24_ on Day 1 are shown for durlobactam and sulbactam in Fig. 4 and 5, respectively, for the following subject types relative to a reference population [i.e., subjects with HABP infection who are not from an East Asian region, have a body weight of 75 kg, and have normal renal function (CLcr of 100 mL/min/1.73 m^2^)]: subjects with augmented renal function (CLcr >130 mL/min/1.73 m^2^), subjects with various degrees of renal impairment, subjects with high or low body weight, subjects from an East Asian region, and subjects with various infection types. Simulated patients received dosing regimens matching those administered in Study CS2514-2017-0004, which included those with augmented renal function receiving sulbactam/durlobactam doses of 1.5 g/1.5 g every 6 hours (q6h).

The greatest differences in exposures were predicted for subjects with CLcr below 30 mL/min/1.73 m^2^ who were expected to have elevated exposures of both durlobactam and sulbactam despite receiving lower daily doses of sulbactam–durlobactam. The implications for this observation in terms of appropriate sulbactam–durlobactam dosing were considered in the context of the PK-PD and PK-PD target attainment analyses (20, 21). Subjects with extreme body weight were also predicted to have somewhat large deviations in exposures. However, the median deviations were within 200% of the median for subjects with normal body weight, suggesting that the differences were unlikely to be clinically relevant. The impact of infection type and East Asian region (defined as patients from China. Taiwan, or South Korea) was minimal, with median deviations within 200%, and thus, these patient factors are unlikely to be clinically relevant.

## DISCUSSION

The primary objective of the analyses was to develop a combined population PK model for sulbactam–durlobactam using six Phase 1 studies, a Phase 2 study (Study CS2514-2017-0003), and a Phase 3 study (Study CS2514-2017-0004). A key facet of the development of the model was the identification of subject-specific factors associated with the IIV in sulbactam–durlobactam PK. The model was then qualified to demonstrate it was sufficiently robust to estimate exposures in the Phase 3 patients and that model-based simulations would reliably predict PK in future patients. The model-predicted exposures from Phase 2 and 3 patients were used to explore potential differences in PK among various subgroups. In addition, sub-models were constructed to describe the impact of HD on the PK of sulbactam–durlobactam and describe the expected time-course of sulbactam–durlobactam in ELF after sulbactam–durlobactam administration.

The data set used to construct the model was large and diverse. A total of 373 subjects provided 5,390 plasma concentrations, including 110 patients (595 concentration observations) with infections due to ABC who had been enrolled in the Phase 3 study. Intensive PK data from six subjects from the renal impairment study (CS2514-2017-0002) were available for the development of the HD sub-models. Observed concentrations in ELF from healthy subjects enrolled in Study CS2514-2017-0001 (*n* = 30, one ELF sample assayed for both analytes from each subject) were available for the development of the ELF sub-models.

Overall, the final four-compartment (two compartments per drug) model with linear kinetics indicated that there was moderate IIV in sulbactam PK as the IIV estimates range from 30.4% for Vc to 46.0% for CL. The IIV in durlobactam was lower than that for sulbactam, with all three IIV terms being close to 28%. While a single additive plus proportional RV model was implemented for sulbactam across all three study phases, it was necessary to fit separate RV models by study phase for durlobactam to account for the higher RV seen in Phase 2 and 3 patients.

Examination of the predicted sulbactam–durlobactam exposures in various patient subgroups (i.e., renal function, age, weight, and sex) indicated that the renal function is likely to be the most clinically relevant covariate. This was seen in both the *post hoc* PK estimates and the model-based simulations. This was expected as previous population PK analyses were used to inform dose adjustments in patients with augmented renal function and severe renal impairment for the Phase 3 study protocol (11). However, the examination of the *post hoc* exposures in patients with severe renal impairment suggests that further dose reductions may be warranted in these subjects. The discrepancy between the predictions from the previous model and the current model was likely a consequence of the additional data that were available for the current analyses. This additional data resulted in a significant change to the population PK model, in which a parameter was added to account for the fact that CL was lower in subjects with CLcr less than 30 mL/min/1.73 m^2^ than expected based upon the original CL:CLcr relationship alone. The implications of this finding, along with an evaluation of appropriate sulbactam–durlobactam dosing regimens for patients with severe renal impairment (i.e., CLcr below 30 mL/min/1.73 m^2^), were evaluated in the context of the results of PK-PD target attainment analyses for efficacy (20, 21).

One other covariate relationship that may have implications for sulbactam–durlobactam dosing was the observation that subjects with extreme body weight are also predicted to have large deviations in exposures relative to those with normal body weight (75 kg). This observation came from the model-based simulations as the number of subjects with extreme body size in the Phase 2 and 3 studies was relatively low. The fact that the model-based simulations showed median deviations for the high and low body weight subjects within 200% of the median for subjects with normal body weight suggested that body weight is not a clinically significant covariate. The implications of the predicted deviations in exposures for subjects with very high body weights were evaluated as part of the PK-PD target attainment analyses (20, 21).

The two other patient factors that were associated with the IIV in PK for sulbactam–durlobactam were infection type and East Asian region. Despite being statistically significant, the magnitude of the impact of these two covariates on PK was minimal. This was supported by the evaluation of *post hoc* PK estimates and the model-based simulations. Ultimately, these factors are unlikely to be clinically relevant.

Overall, the qualification of the final population PK model indicated that the primary objective of the analyses was met. A robust description of the plasma PK of sulbactam–durlobactam in healthy subjects and infected patients was achieved, such that the derived measures of plasma exposures were expected to be both accurate and precise. The resultant population PK model was expected to be appropriate for model-based simulations and assessment of PK-PD relationships for sulbactam–durlobactam (20). In addition, the HD sub-model allowed for the quantification of the impact of HD on CL of both durlobactam and sulbactam while the ELF sub-model supported the development of sulbactam–durlobactam by facilitating the estimation of PK-PD target attainment in ELF (20, 21).

## MATERIALS AND METHODS

Data were obtained from six Phase 1 studies, one Phase 2 study in patients with cUTI/AP, and one Phase 3 study in patients with infections caused by ABC. Phase 1 studies include a SAD/MAD study, an ELF study, a renal impairment study, an excretion and metabolism study, a thorough QT study, and a study in Chinese subjects. Detailed study descriptions including PK sampling times can be found in Table S1.

### Bioanalytical assay

Durlobactam and sulbactam concentrations in human plasma and bronchoalveolar lavage (BAL) fluid were determined using a liquid chromatography-tandem mass spectrometry method. The lower limit of quantitation (LLOQ) was 5 ng/mL in plasma for both durlobactam and sulbactam and 2 ng/mL in BAL fluid for both durlobactam and sulbactam. An assay for urea was utilized to estimate the volume of ELF recovered in the BAL and determine ELF concentrations of durlobactam and sulbactam (13).

For Study CS21514-2017-0004, the LLOQ for the durlobactam plasma assay was the same as above. However, the sulbactam LLOQ was 30 ng/mL for the Chinese sites and 10 ng/mL for sites outside of mainland China. For Study ZL-2402-001, the LLOQ was 10 ng/mL for durlobactam and 30 ng/mL for sulbactam (16).

### Handling of missing data, outliers, and samples determined to be below the limit of quantitation

Specific durlobactam or sulbactam plasma concentrations were excluded from the population PK analyses for any of the following reasons: the date or time of the previous dose was missing, the date or time of sample collection was missing, drug concentration values were determined to be outliers, or the required covariate information (e.g., demography or laboratory) was missing.

BLQ concentration values were flagged in the data set and ignored by the analysis program. In all data presentations (except listings), BLQ concentrations were set to 0.

Outliers were excluded from these analyses given the potential for these observations to negatively impact model convergence and/or parameter estimation. An outlier was defined as an aberrant observation that substantially deviated from the rest of the observations within an individual or across all study subjects.

### Population pharmacokinetic modeling

The population PK analyses were conducted using NONMEM software Version 7.4 (ICON Development Solutions, Ellicott City, MD) implementing the first-order conditional estimation method with interaction (23).

The development of the population PK model involved three main steps: (i) construction of the base structural/statistical models, (ii) conduct of a covariate analysis to identify subject factors associated with the IIV in PK, and (iii) evaluation and qualification of the final model. Base structural model construction consisted of the fitting of separate population PK models, which had been developed previously (11), to the pooled plasma data from the Phase 1, 2, and 3 studies (Fig. S18). The base structural models were then refined separately, as necessary, to assure a robust fit to data from Phase 1 subjects and infected patients. After appropriate base structural models were identified, population PK covariate model development was undertaken for each analyte using forward selection (α = 0.01) followed by a backward elimination (α = 0.001) procedure. The resultant final population PK models were then evaluated for potential revisions such as the removal of extraneous covariate relationships or modifications to the IIV and RV models. The separate models were then combined into one consolidated model in which durlobactam and sulbactam concentrations were fit simultaneously. The model was then qualified by performing a PC-VPC, which graphically examines the agreement between the 5th, 50th, and 95th percentiles of the observed and the individual simulated concentrations across time intervals. The PC-VPC plots were generated using PERL-speaks NONMEM (PsN) for the simulations (*n* = 1,000) and the “vpc” package for R developed by Ron Keizer to generate the images (http://vpc.ronkeizer.com/). In addition, the precision of the PK parameters for the final model was evaluated using an SIR procedure (Fig. S19) (24, 25).

Once the final plasma population PK model was constructed, the HD and ELF sub-models were constructed. The approach taken to develop both sub-models was to use the final model parameters and fix them to their population mean values and only estimate the parameters describing either the impact of HD (for the HD sub-model) or the time-course of ELF concentrations (for the ELF sub-model).

The final step in the analyses was to derive estimates of sulbactam–durlobactam plasma exposures for each subject using the predicted concentration–time profiles, which were generated from the Bayesian PK parameter estimates obtained from the final population PK model. This was accomplished by transitioning the NONMEM model code to C++ code so that simulations can be conducted using mrgsolve, a package that facilitates simulations from differential equation-based models (26). The AUC_0–24_ and *C*_max_ on each treatment day were determined. Secondary PK parameters (e.g., half-life) were generated directly from the individual *post hoc* PK parameters. These *post hoc* estimates were used to evaluate the potential for differences in sulbactam–durlobactam exposures across relevant patient sub-groups (e.g., based on renal function, body size, age, etc.). The potential impact of statistically significant covariate relationships was also evaluated using model-based simulations.

## SUPPLEMENTAL MATERIAL

Supplemental material aac.00485-24-s0001.pdf Tables S1 to S13 and Figures S1 to S19.

ASM does not own the copyrights to Supplemental Material that may be linked to, or accessed through, an article. The authors have granted ASM a non-exclusive, world-wide license to publish the Supplemental Material files. Please contact the corresponding author directly for reuse.
