# Supplementary material for: Population pharmacokinetic analyses for sulbactam–durlobactam using Phase 1, 2, and 3 data
Source: Antimicrob Agents Chemother. 2024 Nov 21;69(1):e00485-24. doi: 10.1128/aac.00485-24 (PMC11784299; doi:10.1128/aac.00485-24)

Abbreviations Listing

Abbreviation	Abbreviation definition
AUC_{0-8}	Area under the concentration-time curve from time 0 to 8 hours (mg•h/L)
AUC_{0-12}	Area under the concentration-time curve from time 0 to 12 hours (mg•h/L)
AUC_{0-24}	Area under the concentration-time curve from time 0 to 24 hours (mg•h/L)
AP	Acute pyelonephritis
BAL	Bronchoalveolar lavage
BLQ	Below the limit of quantitation
BMI	Body mass index (kg/m^2)
BSA	Body surface area (m^2)
CIL	Cilastatin
CL	Clearance (L/h)
CLcr	Creatinine clearance (mL/min or mL/min/1.73 m^2)
CLnr	Non-renal clearance (L/h)
CLr	Renal clearance (L/h)
CLt	Total clearance (L/h)
cm	Centimeters
C_{max}	Maximum plasma concentration (mg/L)
cUTI	Complicated urinary tract infection
%CV	Percent coefficient of variation
DOSEMG	Dose in milligrams
DUR	Durlobactam
eGFR	Estimated glomerular filtration rate (mL/min/1.73 m^2)
ELF	Epithelial lining fluid
g	Grams
h	Hours
HABP	Hospital-acquired bacterial pneumonia
HD	Hemodialysis
IMI	Imipenem
INFTYPN	Infection type flag (numeric)
kg	Kilograms
L	Liters
m	Meters
Max	Maximum
mg	Milligrams
Min	Minimum
min	Minute
mL	Milliliters
n	Number of subjects/patients or observations

N/A	Not applicable
NPDE	Normalized prediction distribution errors
ω^2	Interindividual variability
PK	Pharmacokinetic
q6h	Every 6 hours
q12h	Every 12 hours
σ^2	Residual variability
SD	Standard deviation
%SEM	Percent standard error of the mean
SUL	Sulbactam
$t_{1/2\alpha}$	Alpha half-life (h)
$t_{1/2\beta}$	Beta half-life (h)
VABP	Ventilator-associated bacterial pneumonia
Vc	Volume of distribution in the central compartment (L)
Vp	Volume of distribution in the peripheral compartment (L)
V_{SS}	Volume of distribution at steady state (L)
yr	Years

Supplementary Tables

Table S1. Study descriptions

Study Number	Study Title	Phase	Number of Subjects/Patients[a]	Dosing Scheme[b]	PK Sampling Times[c]
CS2514-2016-0001 [1]	A Phase I, double-blind, randomized, placebo-controlled study to evaluate the safety, tolerability, and pharmacokinetics of intravenous ETX2514 administered in healthy subjects	1	Part A: 64<br>Part B: 32<br>Part C: 16<br>Part D: 12	Part A: single doses of 0.25, 0.5, 1, 2, 4, or 8 g DUR<br><br>Part B: single doses of 0.25, 0.5, 1, and 2 g DUR q6h for 7 days with 1 dose on Day 8<br><br>Part C: single doses of 1 g DUR, 1 g DUR with 1 g SUL, 1 g SUL; or 1 g DUR, 0.5 g IMI-CIL, 1 g DUR with 0.5 g IMI-CIL, and 1 g DUR with 1 g Sul and 0.5 g IMI-CIL in crossover fashion<br><br>Part D: 1 g DUR with 1 g SUL and 0.5 g IMI-CIL, or 1 g SUL with 0.5 g IMI-CIL, all q6h for 10 days and 1 dose on Day 11	Part A: pre-dose and 1, 2, 3, 3.5, 4, 5, 6, 8, 12, 24, 36, and 48 h for the 3-h infusions. For the 2-h infusions, the 3.5-h sample was collected at 2.5 h.<br><br>Part B: Days 1 and 8 pre-dose and 1, 2, 3, 3.5, 4, 5, 6, 8, 12 h; prior to 2nd dose on Days 2 and 4; Day 9 at 24 and 36 h after final dose<br><br>Part C: pre-dose and 1, 2, 3, 3.5, 4, 5, 6, 8, 12, 24, 36, 48 h after each period<br><br>Part D: Days 1 and 11 pre-dose and 1, 2, 3, 3.5, 4, 5, 6, 8, and 12h; prior to 2nd dose on Days 2 and 4; and 24, 36, and 48 h after final infusion
CS2514-2017-0001 [2]	A Phase I study to determine and compare plasma, epithelial lining fluid, and alveolar macrophage concentrations of intravenous ETX2514 and sulbactam administered to healthy adult subjects	1	30	1 g DUR with 1 g SUL q6h for 3 doses	Pre-dose and 1, 2, 2.5, 2.95, 3.05, 3.25, 3.5, 4, 5 and 6 h after 3rd dose<br><br>BAL:1, 2.5, 3.25, 4, or 6 h
CS2514-2017-0002 [3]	Evaluation of the pharmacokinetics, safety, and tolerability of intravenous ETX2514 and sulbactam administered concurrently to subjects with various	1	34	Cohorts 1-3[d]: 1 g DUR with 1 g SUL<br><br>Cohorts 4 and 5[d,e]: 0.5 g DUR with 0.5 g SUL	Cohorts 1-4: pre-dose and 0.5, 1, 2, 3, 3.5, 4, 5, 6, 7, 8, 12, 24, 36, and 48 h. Additional sample at 60 h for Cohorts 3 and 4.

Study Number	Study Title	Phase	Number of Subjects/Patients[a]	Dosing Scheme[b]	PK Sampling Times[c]
	degrees of renal impairment and healthy matched control subjects				Cohort 5: pre-dose and 0.5, 1, 2, 3, 3.5, 4, 5, 6, 7, 8, 10, 12, 16, 24, 36, 48, and 60 h
CS2514-2018-0002 [4]	Single period, open-label, Phase 1 study to determine the excretion and metabolism of ^{14}C-ETX2514 administered intravenously in healthy, male subjects	1	8	Single dose of 1 g non-labeled DUR	Pre-dose and 1, 2, 3, 4, 6, 8, 10, 12, 18, 24, 36, 48, 72, 96, 120, 144, and 168 h
CS2514-2018-0003 [5]	A randomized, three period crossover thorough QT (TQT) study evaluating the effect of ETX2514 on cardiac repolarization in healthy male or female volunteers	1	32	Single dose of 4 g DUR or 400 mg oral moxifloxacin	Pre-dose and 1.5, 3, 3.25, 3.5, 4, 5, 6, 7, 8, 12, and 24 h
ZL-2402-001 [6]	A Phase I, open-label clinical study to evaluate the pharmacokinetics, safety and tolerability of single intravenous administration of sulbactam-ETX2514	1	12	Single dose of 1 g DUR and 1 g SUL	Pre-dose and 1, 2, 3, 3.5, 4, 5, 6, 8, 12, 24, 36, and 48 h
CS25140 2017-0003 [7]	A double-blind, randomized, placebo-controlled study to evaluate the safety and efficacy of intravenous sulbactam-ETX2514 in the treatment of hospitalized adults with complicated urinary tract infections, including acute pyelonephritis	2	80	1 g DUR with 1 g SUL and 0.5 g IMI-CIL q6h for 7 days or 14 days if indicated[f]	Day 1 pre-dose and Day 4 at 3, 3.5, 5, and 6 h
CS2514-2017-0004 [8]	A randomized, active-controlled study to evaluate the efficacy and safety of intravenous sulbactam-ETX2514 in the treatment of patients with infections caused by *Acinetobacter baumannii-calcoaceticus* complex	3	Part A: 177<br>Part B: 28[g]	Part A: 1 g DUR with 1 g SUL and 1 g IMI-CIL q6h or 2.5 mg/kg colistin q12h plus 1 g IMI-CIL q6h for 7-14 days[f]<br><br>Part B: 1 g DUR with 1 g SUL and 1 g IMI-CIL q6h for 7-14 days[f]	Intensive sampling: Day 1 at 3, 4.5, and 6 h; Day 4 at 3 and 6 h[h]<br><br>Sparse sampling: pre-dose on Days 1 and 4

Note: Abbreviations are provided in the **Abbreviations Listing** on Pages 1-2.

a. Includes all subjects who received at least 1 dose of study treatment, including placebo.

Study Number	Study Title	Phase	Number of Subjects/Patients[a]	Dosing Scheme[b]	PK Sampling Times[c]

b. All medications administered as IV infusions unless otherwise noted. DUR and SUL were administered as 3-hour infusions in all cases except one cohort in Part A of Study CS2514-2017-0001 in which subjects received 1 g DUR over 2 hours. IMI-CIL was infused over 30 minutes in all studies except CS2514-2017-0004, where it was infused over 1 hour. Colistin was infused over 30 minutes.
c. All samples were collected from plasma unless otherwise noted. Times are relative to the start of infusion.
d. Cohort 1 was defined as CLcr ≥ 90 mL/min/1.73 m^2 (normal renal function); Cohort 2, eGFR ≥ 60 to < 90 mL/min/1.73 m^2 (mild renal impairment); Cohort 3, ≥ 30 to < 60 mL/min/1.73 m^2 (moderate renal impairment); Cohort 4, < 30 mL/min/1.73 m^2 (severe renal impairment); Cohort 5, end-stage renal disease on hemodialysis.
e. Cohort 5 was dosed twice: once post-hemodialysis and once pre-hemodialysis.
f. Dose adjustment allowed for renal impairment.
g. Includes two patients from Part A.
h. Intensive sampling was only employed for the first 30 patients in Part A. Day 1 samples were collected after patients had at least two infusions.

References

1. Lickliter JD, Lawrence K, O'Donnell J, Isaacs R. Safety, pharmacokinetics, and drug-drug interaction potential of intravenous durlobactam, a β-lactamase inhibitor, in healthy subjects. Antimicrob Agents Chemother 2020;64:e00071-20.
2. Rodvold KA, Gotfried MH, Isaacs RD, O'Donnell JP, Stone E. Plasma and intrapulmonary concentrations of ETX2514 and sulbactam following intravenous administration of ETX2514SUL to healthy adult subjects. Antimicrob Agents Chemother 2018;62:e01089-18.
3. O'Donnell J, Preston RA, Mamikonyan G, Stone E, Isaacs R. Pharmacokinetics, safety, and tolerability of intravenous durlobactam and sulbactam in subjects with renal impairment and healthy matched control subjects. Antimicrob Agents Chemother 2019;63:e00794-19.
4. Study to determine the excretion and metabolism of 14C-ETX2514 administered intravenously in healthy male subjects. ClinicalTrials.gov identifier: NCT04018950. Updated August 30, 2019. https://clinicaltrials.gov/study/NCT04018950. Accessed on August 14, 2024.
5. O'Donnell J, Maloney K, Steidler M, Morrison R, Isaacs R. A randomized, double-blind, placebo- and positive-controlled crossover study of the effects of durlobactam on cardiac repolarization in healthy subjects. Clin Transl Sci 2021;14:1423-1430.
6. Data on file. Entasis Therapeutics, Inc., an affiliate of Innoviva Specialty Therapeutics, Inc., Waltham, MA.
7. Sagan O, Yakubsevitch R, Yanev K, Fomkin R, Hines D, O'Donnell J, Miller A, Isaacs R, Srinivasan S. Pharmacokinetics and tolerability of intravenous sulbactam-durlobactam with imipenem-cilastatin in hospitalized adults with complicated urinary tract infections, including acute pyelonephritis. Antimicrob Agents Chemother 2020;64:301506-19.
8. Kaye KS, Shorr AF, Wunderink RG, Poirier GE, Rana K, Miller A, Lewis D, O'Donnell J, Chen L, Reinhart H, Srinavasan S, Isaacs R, Altarac D. Efficacy and safety of sulbactam-durlobactam versus colistin for the treatment of patients with serious infections caused by Acinetobacter baumannii-calcoaceticus complex: a multicenter, randomized, active-controlled, phase 3, non-inferiority clinical trial (ATTACK) [published online ahead of print, 2023 May 11]. Lancet Infect Dis. 2023;S1473-3099(23)00184-6.

Table S2. Description of data excluded from the population PK analyses

Study	Matrix	Subjects[a]/ records at start	BLQ samples excluded[b]	Missing samples excluded	Samples without time excluded	Out of protocol samples excluded	Pre-dose samples excluded	Not done samples excluded	Subjects/Records at end
ZL-2402-001	Plasma	12/312	85	0	0	0	0	0	12/227
CS2514-2016-0001	Plasma	114/3268	1322	0	10	0	2	0	96/1934
CS2514-2017-0001	ELF	30/60	0	0	0	0	0	0	30/60
	Plasma	30/780	59	0	0	0	1	0	30/720
CS2514-2017-0002	Plasma	34/1302	180	0	0	0	2	3	34/1117
CS2514-2017-0003	Plasma	68/670	253	0	0	0	1	0	52/416
CS2514-2017-0004	Plasma	135/770	8	140	0	14	2	0	112/606
CS2514-2018-0002	Plasma	8/144	66	0	0	0	0	0	8/78
CS2514-2018-0003	Plasma	31/854	508	6	0	0	0	0	31/340
**Total Plasma[c]**		432/8100	2481	146	10	14	8	3	375/5438

Note: Abbreviations are provided in the **Abbreviations Listing** on Pages 1-2. For this table, “records” are individual concentration observations, not plasma samples.

a. Number of subjects or patients included in the PK population of each study. Number of subjects/patients includes those who received placebo.

b. Plasma BLQ samples and outlier observations were retained in the dataset but flagged so that they were ignored by NONMEM. It should be noted that 1576 of the 2481 BLQ samples in the dataset were either drawn prior to a sulbactam-durlobactam dose or were drawn after placebo/control.

c. Does not include ELF samples.

Table S3. Summary statistics or counts of the subject characteristics for the PK analysis population

Variable	Phase 1						Phase 2	Phase 3	Pooled Dataset (n = 373)
	ZL-2402-001	CS2514-2016-0001	CS2514-2017-0001	CS2514-2017-0002	CS2514-2018-0002	CS2514-2018-0003	CS2514-2017-0003	CS2514-2017-0004	
Age (yr)	28.4 (26.7) 28.5 (19-41)	30.3 (41.6) 26.5 (18-74)	41.7 (26.5) 44 (21-55)	59.5 (15.7) 60.5 (34-71)	36.2 (28.2) 36.5 (19-54)	34.1 (28.4) 32 (20-54)	51.7 (34) 52.5 (18-82)	62.1 (25.1) 64.5 (18-91)	46.6 (41.2) 46 (18-91)
Height (cm)	166 (5.01) 170 (147-176)	174 (5.3) 175 (153-195)	173 (5.03) 173 (154-188)	169 (5.06) 171 (149-186)	176 (4.04) 177 (166-185)	170 (5.02) 170 (153-190)	173 (5.15) 173 (155-198)	170 (5.32) 170 (145-192)	171 (5.31) 171 (145-198)
Weight (kg)	57.6 (11.4) 58 (47.5-68.2)	72.6 (17.4) 72.8 (44.9-104)	79.7 (14.8) 81.7 (61.2-97.1)	86.2 (16.2) 85.9 (60-117)	81.2 (14.2) 79.8 (67.6-98.7)	73.8 (14.2) 74.1 (52.5-93.9)	83.6 (24.9) 82.8 (49.5-135)	75 (26.6) 75 (35.8-150)	76.4 (22.3) 75 (35.8-150)
BSA (m^2)	1.64 (8.08) 1.69 (1.38-1.8)	1.87 (10.5) 1.88 (1.39-2.26)	1.94 (9.5) 1.93 (1.6-2.24)	1.96 (9.14) 1.98 (1.65-2.31)	1.97 (8.2) 1.96 (1.79-2.17)	1.85 (8.87) 1.86 (1.52-2.14)	1.96 (12.5) 1.98 (1.52-2.45)	1.85 (13.6) 1.85 (1.21-2.69)	1.88 (11.8) 1.87 (1.21-2.69)
BMI (kg/m^2)	20.8 (6.71) 20.4 (19.1-23.4)	23.8 (12.7) 23.5 (19-31.8)	26.5 (9.36) 26.8 (19.3-30.2)	30.2 (15.3) 29.7 (22.3-40.3)	26.2 (10.9) 25.9 (22.3-30.9)	25.6 (11.1) 25.7 (19.8-29.6)	28 (23.9) 27.4 (18.4-52.1)	25.9 (22) 25.7 (11-44.8)	25.9 (19.5) 25.7 (11-52.1)
CLcr (mL/min/1.73 m^2)	116 (10) 115 (99.6-141)	1156(18) 117 (56.1-164)	81.5 (17.5) 79.3 (47.9-112)	38.4 (70.1) 35.3 (5.61-85.5)	95.5 (14) 97.4 (71.6-115)	87.8 (15.8) 86.9 (61.9-116)	74.5 (33.6) 70.1 (26-139)	95 (63.7) 85.2 (10.5-364)	91.3 (47.2) 91.5 (5.61-364)
Sex									
Male	6 (50)	59 (61.5)	18 (60)	18 (52.9)	8 (100)	16 (51.6)	26 (50)	82 (74.5)	233 (62.5)
Female	6 (50)	37 (38.5)	12 (40)	16 (47.1)		15 (48.4)	26 (50)	28 (25.5)	140 (37.5)
Country of Origin									
China	12 (100)							23 (20.9)	35 (9.38)
Other		96 (100)	30 (100)	34 (100)	8 (100)	31 (100)	52 (100)	87 (79.1)	338 (90.6)
Region									
East Asia	12 (100)							33 (30)	45 (12.1)
Other		96 (100)	30 (100)	34 (100)	8 (100)	31 (100)	52 (100)	77 (70)	328 (87.9)
Race									
White		74 (77.1)	17 (56.7)	23 (67.6)	2 (25)	10 (32.3)	52 (100)	66 (60)	244 (65.4)
Black		3 (3.12)	9 (30)	11 (32.4)	5 (62.5)	21 (67.7)			49 (13.1)

Variable	Phase 1						Phase 2	Phase 3	Pooled Dataset (n = 373)
	ZL-2402-001	CS2514-2016-0001	CS2514-2017-0001	CS2514-2017-0002	CS2514-2018-0002	CS2514-2018-0003	CS2514-2017-0003	CS2514-2017-0004	
Asian	12 (100)	12 (12.5)	1 (3.33)					35 (31.8)	60 (16.1)
American Indian/ Alaska Native			1 (3.33)		1 (12.5)			5 (4.55)	7 (1.88)
Other		7 (7.29)	2 (6.67)					4 (3.64)	13 (3.49)

Note: Abbreviations are provided in the **Abbreviations Listing** on Pages 1-2. Summary statistics presented as mean (CV%) and median (Min. – Max.) or n/N (%).

Table S4. Parameter estimates and their associated precision (%SEM) for the HD sub-models fit to Study CS2514-2017-0002 data

Parameter	Final estimate	%SEM	Shrinkage
CL-HDEFFECT (durlobactam)	6.24	14.5	N/A
CL-HDEFFECT (sulbactam)	8.19	23.2	
$\omega^2_{CL\text{-}HDEFFECT}$ (durlobactam)	0.124 (35.2 %CV)	40.9	57.8
$\omega^2_{CL\text{-}HDEFFECT}$ (sulbactam)	0.316 (56.2 %CV)	52.3	58.1

Note: Abbreviations are provided in the **Abbreviations Listing** on Pages 1-2.

Table S5. Summary statistics (geometric mean [CV%]) for key durlobactam and sulbactam exposures for subjects with severe renal impairment receiving hemodialysis

Parameter	Durlobactam (n = 6)		Sulbactam (n = 6)	
	Period 1 (after HD)	Period 2 (before HD)	Period 1 (after HD)	Period 2 (before HD)
$AUC_{0\text{-}8}$ (mg•h/L)	135 (20.8%)	93.3 (20.5%)	117 (29.4%)	93.0 (33.9%)
$AUC_{0\text{-}12}$ (mg•h/L)	181 (25.8%)	104 (21.5%)	163 (36.8%)	104 (33.9%)
$AUC_{0\text{-}24}$ (mg•h/L) with HD[a]	299 (24.5%)		271 (34.2%)	
$AUC_{0\text{-}24}$ (mg•h/L) without HD[b]	435 (30.9%)		410 (44.9%)	
C_{max} (mg/L)	25.4 (15.6%)	25.4 (15.0%)	21.5 (20.1%)	21.5 (20.1%)

Note: Abbreviations are provided in the **Abbreviations Listing** on Pages 1-2.

a. Calculated by simulating the concentration profile for 24 hours with two 500 mg/500 mg sulbactam-durlobactam doses given 12 hours apart with intermittent HD started one hour after the end of the morning infusion.

b. Calculated by simulating the concentration profile for 24 hours with two 500 mg/500 mg sulbactam-durlobactam doses given 12 hours apart and no HD.

Table S6. Parameter estimates and their associated precision (%SEM) for the ELF sub-models fit to Study CS2514-2017-0001 data

Parameter	Final estimate	%SEM	Shrinkage
PLASMA-ELF ratio (durlobactam)	0.372	3.6	N/A
PLASMA-ELF ratio (sulbactam)	0.533	5.41	
$\sigma^2_{ELF, Proportional}$ (durlobactam)	0.0322 (17.9 %CV)	31.8	3.91
$\sigma^2_{ELF, Proportional}$ (sulbactam)	0.0628 (25.1 %CV)	32.6	2.56

Note: Abbreviations are provided in the **Abbreviations Listing** on Pages 1-2.

Table S7. Summary statistics (geometric mean [CV%]) for key durlobactam and sulbactam exposures in ELF and plasma for subjects enrolled in the ELF penetration study (Study CS2514-2017-0001)

Parameter	Durlobactam (n = 30)		Sulbactam (n = 30)	
	ELF	Plasma	ELF	Plasma
AUC_{0-24} (mg•h/L)	118 (19.2%)	316 (19.2%)	103 (21.7%)	193 (21.7%)
C_{max} (mg/L)	11.0 (17.6%)	29.5 (17.6%)	10.2 (19.9%)	19.1 (19.9%)

Note: Abbreviations are provided in the **Abbreviations Listing** on Pages 1-2.

Table S8. Summary statistics (geometric mean [CV%]) for key durlobactam and sulbactam exposures and PK parameters for subjects enrolled in the Phase 2 and 3 studies, stratified by CLcr group in mL/min

Parameter	CLcr group					
	**0-14 mL/min (n = 2)**	**15-29 mL/min (n = 12)**	**30-59 mL/min (n = 26)**	**60-89 mL/min (n = 50)**	**90-129 mL/min (n = 37)**	**≥130 mL/min (n = 35)**
**Durlobactam**						
CLcr (mL/min)	10.8 (1.31%)	21.0 (17.3%)	44.0 (21.1%)	74.4 (11.9%)	107 (11.7%)	175 (26.5%)
AUC_{0-24}, Day 1 (mg•h/L)	649 (31.3%)	637 (28.0%)	600 (35.1%)	425 (31.5%)	409 (37.7%)	406 (38.5%)
AUC_{0-24}, Day 2 (mg•h/L)	842 (54.9%)	976 (28.6%)	579 (96.9%)	467 (32.6%)	449 (40.2%)	443 (42.6%)
AUC_{0-24}, Day 3 (mg•h/L)	440 (159%)	996 (35.0%)	410 (212%)	453 (34.8%)	418 (53.5%)	400 (90.6%)
C_{max}, Day 1 (mg/L)	45.3 (31.5%)	43.5 (26.9%)	38.6 (32.0%)	28.4 (31.0%)	27.5 (36.4%)	27.3 (39.1%)
C_{max}, Day 2 (mg/L)	54.3 (37.4%)	51.6 (26.4%)	34.6 (67.0%)	28.9 (29.8%)	28.0 (37.2%)	27.7 (39.3%)
C_{max}, Day 3 (mg/L)	28.5 (136%)	53.9 (27.0%)	24.6 (182%)	28.9 (29.8%)	27.0 (39.3%)	25.7 (67.4%)
CL (L/h)	1.83 (35.2%)	2.97 (20.4%)	5.43 (37.7%)	8.60 (32.9%)	9.40 (36.6%)	12.0 (41.7%)
Vc (L)	25.5 (34.7%)	34.6 (44.9%)	23.5 (34.5%)	20.9 (48.4%)	21.6 (51.9%)	31.8 (48.6%)
Vss (L)	31.5 (28.3%)	40.9 (38.1%)	29.5 (28.1%)	27.1 (39.1%)	28.0 (40.3%)	38.1 (41.1%)
$t_{1/2,\alpha}$ (h)	0.732 (7.12%)	0.761 (8.97%)	0.675 (9.77%)	0.606 (17.9%)	0.601 (21.8%)	0.677 (16.6%)
$t_{1/2,\beta}$ (h)	12.1 (7.22%)	9.70 (26.5%)	4.02 (31.5%)	2.51 (30.7%)	2.41 (29.9%)	2.44 (32.2%)
**Sulbactam**						
CLcr (mL/min)	10.8 (1.31%)	21.0 (17.3%)	44.0 (21.1%)	74.4 (11.9%)	107 (11.7%)	175 (26.5%)
AUC_{0-24}, Day 1 (mg•h/L)	914 (59.3%)	690 (29.1%)	714 (40.4%)	421 (40.2%)	391 (58.2%)	399 (59.3%)
AUC_{0-24}, Day 2 (mg•h/L)	1320 (88.4%)	1050 (37.2%)	733 (89.0%)	458 (43.3%)	423 (62.5%)	436 (68.6%)
AUC_{0-24}, Day 3 (mg•h/L)	783 (187%)	1090 (39.1%)	552 (186%)	445 (43.2%)	391 (76.1%)	406 (91.5%)
C_{max}, Day 1 (mg/L)	61.5 (56.9%)	47.4 (26.9%)	46.2 (37.7%)	29.0 (39.4%)	27.4 (55.3%)	27.9 (55.8%)
C_{max}, Day 2 (mg/L)	79.7 (70.5%)	56.1 (32.5%)	42.9 (62.9%)	29.5 (38.4%)	27.8 (55.5%)	28.2 (58.7%)
C_{max}, Day 3 (mg/L)	47.2 (162%)	59.0 (31.8%)	32.3 (158%)	29.5 (38.6%)	26.7 (59.1%)	27.5 (67.1%)
CL (L/h)	1.07 (80.2%)	2.73 (33.5%)	4.37 (51.7%)	8.76 (44.6%)	9.98 (59.7%)	12.2 (71.2%)
Vc (L)	18.7 (57.9%)	29.7 (38.0%)	18.5 (41.5%)	15.1 (73.4%)	13.9 (90.0%)	25.1 (45.2%)
Vss (L)	25.0 (48.6%)	37.0 (32.3%)	25.9 (24.5%)	22.4 (54.1%)	22.0 (60.8%)	32.3 (36.9%)
$t_{1/2,\alpha}$ (h)	0.402 (29.3%)	0.494 (17.4%)	0.404 (23.6%)	0.332 (45.8%)	0.298 (61.7%)	0.415 (22.5%)
$t_{1/2,\beta}$ (h)	16.4 (31.4%)	9.54 (39.1%)	4.35 (48.2%)	2.04 (50.8%)	1.88 (53.7%)	2.04 (57.6%)

Note: Abbreviations are provided in the **Abbreviations Listing** on Pages 1-2.

Table S9. Summary statistics (geometric mean [CV%]) for key durlobactam and sulbactam exposures and PK parameters for subjects enrolled in the Phase 2 and 3 studies, stratified by body weight category

**Parameter**	**Body weight category**					
	**Durlobactam**			**Sulbactam**		
	**35 – 50 kg (n = 10)**	**51 – 90 kg (n = 121)**	**91 – 150 kg (n = 31)**	**35 – 50 kg (n = 10)**	**51 – 90 kg (n = 121)**	**91 – 150 kg (n = 31)**
$AUC_{0\text{-}24}$, Day 1 (mg•h/L)	739 (21.3%)	459 (37.9%)	382 (30.6%)	797 (39.4%)	474 (54.2%)	369 (45.5%)
$AUC_{0\text{-}24}$, Day 2 (mg•h/L)	856 (23.5%)	500 (59.0%)	438 (35.7%)	938 (47.9%)	520 (69.9%)	419 (57.3%)
$AUC_{0\text{-}24}$, Day 3 (mg•h/L)	870 (27.3%)	429 (115%)	444 (36.1%)	958 (50.9%)	457 (109%)	426 (60.0%)
C_{max}, Day 1 (mg/L)	48.0 (21.4%)	30.8 (36.2%)	25.5 (33.0%)	52.5 (39.3%)	32.6 (50.4%)	25.5 (45.3%)
C_{max}, Day 2 (mg/L)	49.4 (23.8%)	30.8 (46.0%)	26.4 (31.4%)	54.6 (42.7%)	32.9 (56.4%)	26.4 (47.9%)
C_{max}, Day 3 (mg/L)	49.7 (24.8%)	27.5 (95.6%)	26.3 (31.5%)	55.1 (43.4%)	30.2 (88.6%)	26.5 (50.8%)
CL (L/h)	4.97 (32.1%)	7.81 (54.9%)	9.91 (46.0%)	4.51 (52.6%)	7.55 (74.0%)	10.3 (69.9%)
Vc (L)	16.4 (35.6%)	23.6 (45.9%)	32.0 (57.2%)	12.6 (62.4%)	17.2 (70.0%)	23.8 (66.9%)
Vss (L)	22.3 (25.4%)	29.8 (36.5%)	38.5 (48.8%)	19.8 (42.7%)	24.7 (50.4%)	32.8 (42.8%)
$t_{1/2,\alpha}$ (h)	0.600 (17.0%)	0.636 (18.9%)	0.685 (15.3%)	0.347 (44.9%)	0.354 (46.2%)	0.401 (35.0%)
$t_{1/2,\beta}$ (h)	3.43 (25.0%)	2.98 (53.1%)	2.98 (49.8%)	3.32 (54.8%)	2.56 (75.0%)	2.53 (68.2%)

Note: Abbreviations are provided in the **Abbreviations Listing** on Pages 1-2.

Table S10. Summary statistics (geometric mean [CV%]) for key durlobactam and sulbactam exposures and PK parameters for subjects enrolled in the Phase 2 and 3 studies, stratified by BMI category

Parameter	BMI category					
	**Underweight (15 – 18.4 kg/m²) (n = 8)**	**Normal Weight (18.5 – 24.9 kg/m²) (n = 58)**	**Overweight (25.0 – 29.9 kg/m²) (n = 63)**	**Obese, Class 1 (30.0 – 34.9 kg/m²) (n = 18)**	**Obese, Class 2 (35.0 – 39.9 kg/m²) (n = 10)**	**Obese, Class 3 (40.0 – 58 kg/m²) (n = 5)**
**Durlobactam**						
AUC_{0-24}, Day 1 (mg•h/L)	683 (46.2%)	507 (32.6%)	424 (38.6%)	429 (39.1%)	345 (32.5%)	397 (22.9%)
AUC_{0-24}, Day 2 (mg•h/L)	789 (50.2%)	575 (40.8%)	446 (68.3%)	477 (47.0%)	389 (30.0%)	507 (32.1%)
AUC_{0-24}, Day 3 (mg•h/L)	807 (52.9%)	535 (52.0%)	383 (138%)	367 (122%)	401 (32.0%)	526 (36.0%)
C_{max}, Day 1 (mg/L)	44.7 (44.7%)	33.8 (30.0%)	28.6 (38.3%)	28.8 (38.2%)	22.3 (33.0%)	25.8 (22.8%)
C_{max}, Day 2 (mg/L)	46.3 (47.0%)	34.6 (33.5%)	28.0 (51.6%)	29.4 (40.5%)	23.3 (30.0%)	28.5 (25.7%)
C_{max}, Day 3 (mg/L)	46.6 (48.3%)	33.9 (35.3%)	24.2 (119%)	24.4 (87.9%)	23.4 (30.0%)	28.2 (30.6%)
CL (L/h)	5.20 (54.0%)	7.08 (52.6%)	8.51 (53.0%)	8.98 (54.3%)	11.6 (40.4%)	7.69 (57.0%)
Vc (L)	16.3 (46.1%)	21.2 (35.2%)	26.1 (49.3%)	24.4 (54.9%)	37.8 (63.3%)	44.9 (56.7%)
Vss (L)	22.4 (34.2%)	27.2 (27.5%)	32.4 (40.0%)	30.9 (43.5%)	44.6 (54.9%)	51.4 (49.4%)
$t_{1/2,\alpha}$ (h)	0.590 (19.0%)	0.624 (16.7%)	0.649 (19.7%)	0.637 (18.2%)	0.715 (14.3%)	0.759 (12.2%)
$t_{1/2,\beta}$ (h)	3.31 (34.3%)	3.02 (51.2%)	2.95 (54.4%)	2.71 (45.0%)	2.88 (32.9%)	4.86 (72.1%)
**Sulbactam**						
AUC_{0-24}, Day 1 (mg•h/L)	726 (59.0%)	508 (46.6%)	442 (55.9%)	416 (57.7%)	369 (68.2%)	411 (28.1%)
AUC_{0-24}, Day 2 (mg•h/L)	851 (68.4%)	580 (57.1%)	465 (74.6%)	468 (71.3%)	421 (78.3%)	513 (49.2%)
AUC_{0-24}, Day 3 (mg•h/L)	874 (71.4%)	541 (68.4%)	409 (125%)	388 (111%)	432 (81.9%)	536 (56.2%)
C_{max}, Day 1 (mg/L)	48.6 (55.6%)	34.5 (42.4%)	30.7 (52.6%)	29.1 (55.7%)	24.9 (68.3%)	27.0 (27.9%)
C_{max}, Day 2 (mg/L)	50.9 (59.3%)	35.5 (46.8%)	30.1 (59.3%)	30.1 (60.9%)	26.2 (67.1%)	30.1 (40.4%)
C_{max}, Day 3 (mg/L)	51.3 (61.0%)	34.8 (49.9%)	26.7 (107%)	27.3 (75.2%)	26.7 (72.8%)	29.9 (45.7%)
CL (L/h)	4.79 (74.5%)	6.98 (69.2%)	8.26 (71.9%)	9.14 (82.8%)	10.7 (86.4%)	7.47 (76.9%)
Vc (L)	12.2 (71.7%)	16.4 (65.4%)	19.1 (66.4%)	15.3 (90.8%)	27.1 (58.0%)	36.2 (46.1%)
Vss (L)	19.1 (47.7%)	23.9 (46.0%)	26.3 (51.4%)	25.3 (49.5%)	33.9 (51.1%)	43.3 (39.8%)
$t_{1/2,\alpha}$ (h)	0.321 (47.4%)	0.357 (47.1%)	0.369 (42.0%)	0.322 (50.7%)	0.415 (31.3%)	0.474 (18.3%)
$t_{1/2,\beta}$ (h)	3.02 (68.3%)	2.71 (73.1%)	2.47 (71.7%)	2.35 (72.1%)	2.36 (75.4%)	4.19 (92.5%)

Note: Abbreviations are provided in the **Abbreviations Listing** on Pages 1-2.

Table S11. Summary statistics (geometric mean [CV%]) for key durlobactam and sulbactam exposures and PK parameters for subjects enrolled in the Phase 2 and 3 studies, stratified by sex

**Parameter**	**Sex**			
	**Durlobactam**		**Sulbactam**	
	**Female (n = 54)**	**Male (n = 108)**	**Female (n = 54)**	**Male (n = 108)**
AUC_{0-24}, Day 1 (mg•h/L)	479 (36.8%)	446 (39.1%)	502 (53.4%)	449 (54.5%)
AUC_{0-24}, Day 2 (mg•h/L)	518 (58.7%)	497 (54.0%)	548 (61.2%)	502 (72.0%)
AUC_{0-24}, Day 3 (mg•h/L)	454 (115%)	450 (96.3%)	494 (88.2%)	461 (106%)
C_{max}, Day 1 (mg/L)	32.2 (35.0%)	29.7 (38.3%)	35.0 (51.3%)	30.6 (50.7%)
C_{max}, Day 2 (mg/L)	32.3 (44.8%)	30.0 (44.3%)	35.6 (51.8%)	31.1 (58.0%)
C_{max}, Day 3 (mg/L)	29.1 (96.6%)	27.9 (79.1%)	33.1 (70.8%)	29.3 (86.8%)
CL (L/h)	7.51 (46.1%)	8.18 (57.8%)	7.20 (64.8%)	8.05 (78.3%)
Vc (L)	19.8 (51.7%)	27.1 (45.9%)	12.1 (89.3%)	21.9 (48.1%)
Vss (L)	26.1 (40.1%)	33.4 (38.4%)	19.4 (63.0%)	29.5 (34.9%)
$t_{1/2,\alpha}$ (h)	0.593 (22.5%)	0.669 (14.4%)	0.284 (61.0%)	0.409 (25.6%)
$t_{1/2,\beta}$ (h)	2.78 (46.8%)	3.12 (53.0%)	2.20 (74.7%)	2.83 (70.4%)

Note: Abbreviations are provided in the **Abbreviations Listing** on Pages 1-2.

Table S12. Summary statistics [geometric mean (CV%)] for key durlobactam and sulbactam exposures and PK parameters for subjects enrolled in the Phase 2 and 3 studies, stratified by infection type

Parameter	Infection type				
	**cUTI (n = 35)**	**AP (n = 17)**	**Bacteremia (n = 16)**	**HABP (n = 38)**	**VABP (n = 56)**
**Durlobactam**					
$AUC_{0\text{-}24}$, Day 1 (mg•h/L)	405 (29.1%)	463 (39.8%)	400 (42.9%)	520 (33.9%)	466 (42.4%)
$AUC_{0\text{-}24}$, Day 2 (mg•h/L)	431 (31.7%)	484 (42.5%)	522 (49.9%)	563 (64.1%)	517 (64.5%)
$AUC_{0\text{-}24}$, Day 3 (mg•h/L)	431 (31.6%)	484 (42.5%)	546 (52.2%)	452 (133%)	430 (129%)
C_{max}, Day 1 (mg/L)	27.9 (26.5%)	32.9 (36.8%)	25.8 (46.8%)	34.4 (33.0%)	30.5 (40.9%)
C_{max}, Day 2 (mg/L)	27.9 (26.8%)	33.0 (36.9%)	28.0 (45.8%)	33.9 (48.7%)	30.8 (51.2%)
C_{max}, Day 3 (mg/L)	27.9 (26.8%)	33.1 (36.8%)	28.2 (47.5%)	28.9 (111%)	26.9 (107%)
CL (L/h)	9.27 (31.6%)	8.26 (42.5%)	8.93 (59.0%)	6.84 (53.7%)	7.64 (64.7%)
Vc (L)	18.2 (22.3%)	11.5 (29.1%)	59.9 (30.2%)	24.5 (32.6%)	28.6 (29.8%)
Vss (L)	24.1 (16.9%)	17.3 (20.3%)	65.8 (27.4%)	30.4 (26.6%)	34.6 (24.5%)
$t_{1/2,\alpha}$ (h)	0.578 (8.86%)	0.451 (13.7%)	0.806 (6.45%)	0.665 (14.0%)	0.701 (6.75%)
$t_{1/2,\beta}$ (h)	2.14 (20.4%)	1.90 (23.7%)	5.22 (48.8%)	3.36 (48.9%)	3.38 (49.9%)
**Sulbactam**					
$AUC_{0\text{-}24}$, Day 1 (mg•h/L)	352 (34.7%)	504 (57.4%)	427 (70.0%)	482 (51.3%)	545 (54.9%)
$AUC_{0\text{-}24}$, Day 2 (mg•h/L)	366 (36.3%)	518 (61.0%)	546 (84.7%)	528 (80.2%)	622 (66.6%)
$AUC_{0\text{-}24}$, Day 3 (mg•h/L)	367 (36.3%)	519 (61.2%)	569 (88.4%)	424 (149%)	548 (97.8%)
C_{max}, Day 1 (mg/L)	24.9 (31.4%)	38.4 (54.4%)	28.7 (69.4%)	32.3 (47.4%)	36.4 (51.5%)
C_{max}, Day 2 (mg/L)	24.9 (31.6%)	38.6 (54.8%)	30.9 (72.3%)	32.1 (61.4%)	37.4 (55.1%)
C_{max}, Day 3 (mg/L)	24.9 (31.5%)	38.6 (54.8%)	31.3 (76.7%)	27.2 (123%)	34.7 (75.1%)
CL (L/h)	10.9 (36.3%)	7.71 (61.2%)	8.50 (97.3%)	7.20 (76.9%)	6.43 (80.2%)
Vc (L)	15.6 (29.7%)	3.35 (30.1%)	42.3 (28.7%)	24.5 (33.5%)	20.8 (29.0%)
Vss (L)	22.2 (27.2%)	9.09 (45.3%)	49.6 (24.1%)	31.5 (27.0%)	27.9 (23.6%)
$t_{1/2,\alpha}$ (h)	0.344 (22.8%)	0.119 (27.3%)	0.513 (6.63%)	0.433 (18.2%)	0.421 (17.5%)
$t_{1/2,\beta}$ (h)	1.66 (26.2%)	1.24 (55.5%)	4.18 (81.3%)	3.25 (69.5%)	3.23 (67.8%)

Note: Abbreviations are provided in the **Abbreviations Listing** on Pages 1-2.

Table S13. Summary statistics (geometric mean [CV%]) for key exposures and PK parameters for subjects enrolled in the Phase 2 and 3 studies, stratified by study phase and region of origin

Parameter	Study phase by region of origin					
	Durlobactam			Sulbactam		
	Phase 2	Phase 3		Phase 2	Phase 3	
	Non-East Asia (n = 52)	Non-East Asia (n = 77)	East Asia (n = 33)	Non-East Asia (n = 52)	Non-East Asia (n = 77)	East Asia (n = 33)
AUC_{0-24}, Day 1 (mg•h/L)	423 (33.2%)	416 (37.2%)	637 (30.7%)	396 (46.1%)	480 (57.0%)	565 (53.7%)
AUC_{0-24}, Day 2 (mg•h/L)	448 (35.6%)	458 (64.2%)	759 (38.6%)	410 (48.1%)	538 (76.5%)	679 (65.8%)
AUC_{0-24}, Day 3 (mg•h/L)	448 (35.6%)	384 (137%)	667 (64.3%)	411 (48.2%)	468 (127%)	601 (86.4%)
C_{max}, Day 1 (mg/L)	29.4 (30.9%)	27.4 (37.6%)	41.6 (29.0%)	28.6 (44.8%)	32.3 (54.4%)	37.3 (49.9%)
C_{max}, Day 2 (mg/L)	29.5 (31.1%)	27.3 (48.9%)	43.7 (32.9%)	28.7 (45.2%)	32.5 (61.0%)	39.6 (56.0%)
C_{max}, Day 3 (mg/L)	29.5 (31.1%)	23.3 (114%)	41.7 (40.3%)	28.7 (45.2%)	29.0 (105%)	37.8 (62.4%)
CL (L/h)	8.93 (35.6%)	8.71 (55.6%)	5.35 (58.0%)	9.74 (48.2%)	7.46 (78.5%)	5.92 (87.3%)
Vc (L)	15.7 (32.8%)	35.7 (38.5%)	20.3 (16.8%)	9.43 (78.7%)	26.2 (40.9%)	20.7 (26.8%)
Vss (L)	21.6 (23.8%)	41.8 (33.3%)	26.2 (13.1%)	16.6 (54.1%)	33.5 (33.4%)	27.6 (21.1%)
$t_{1/2,\alpha}$ (h)	0.533 (15.7%)	0.727 (11.7%)	0.648 (6.15%)	0.243 (55.8%)	0.445 (19.4%)	0.419 (12.8%)
$t_{1/2,\beta}$ (h)	2.06 (22.0%)	3.55 (52.5%)	3.70 (49.3%)	1.51 (40.2%)	3.32 (70.5%)	3.46 (70.9%)

Note: Abbreviations are provided in the **Abbreviations Listing** on Pages 1-2.

Supplementary Figures

Figure S1. Semi-log plot of plasma concentrations versus time since previous dose, stratified by infection type and paneled by analyte

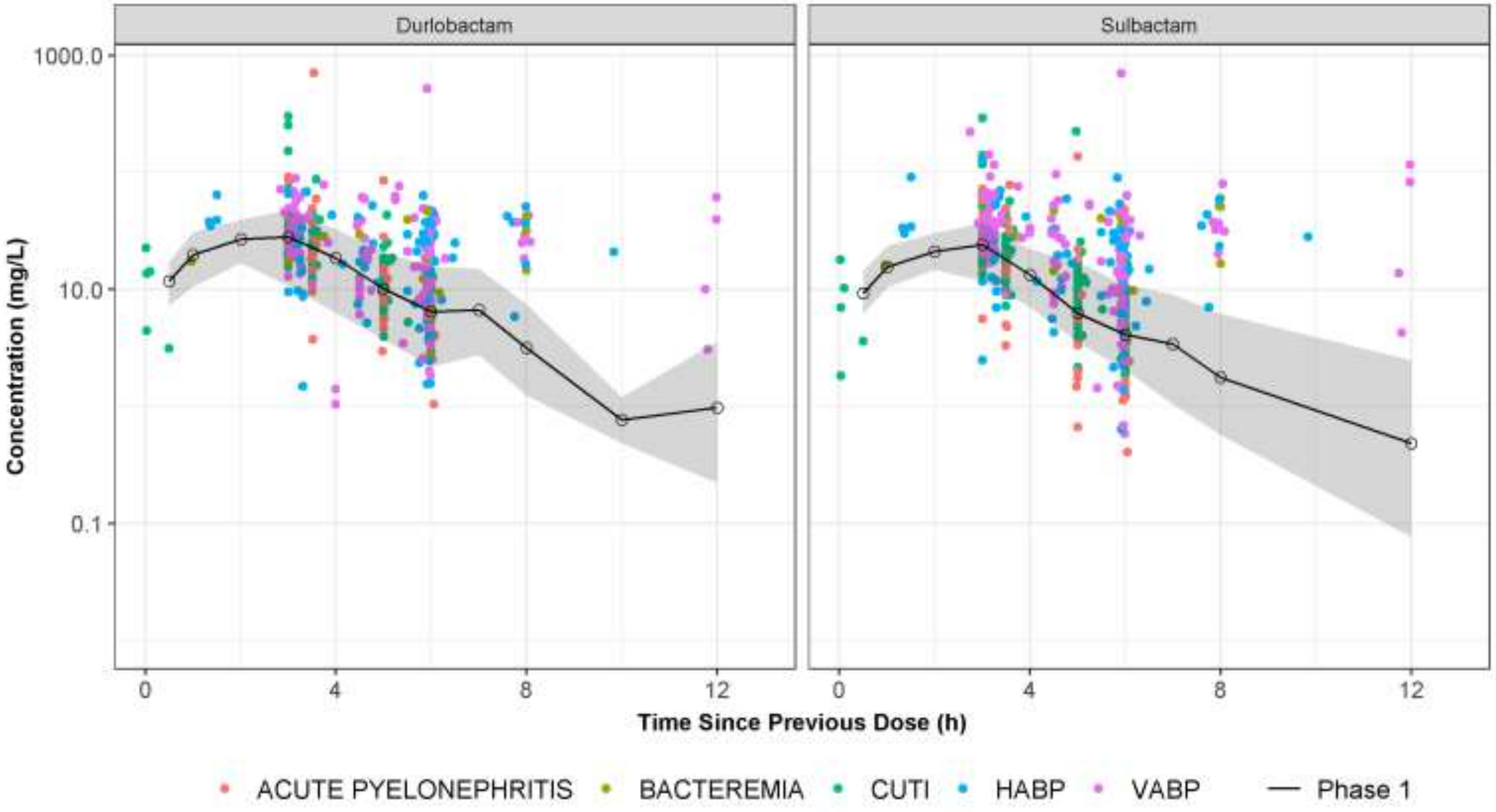

Note: Abbreviations are provided in the **Abbreviations Listing** on Pages 1-2. Solid circles are observed concentrations in infected subjects. Open circles show the mean concentration at each nominal sampling time from Phase 1 subjects with normal renal function receiving 1.0 g sulbactam/1.0 g durlobactam. Line connects the mean values and the grey shaded region shows the 90% confidence interval.

Figure S2. Semi-log plot of plasma concentrations versus time, stratified by analyte and paneled by phase and study

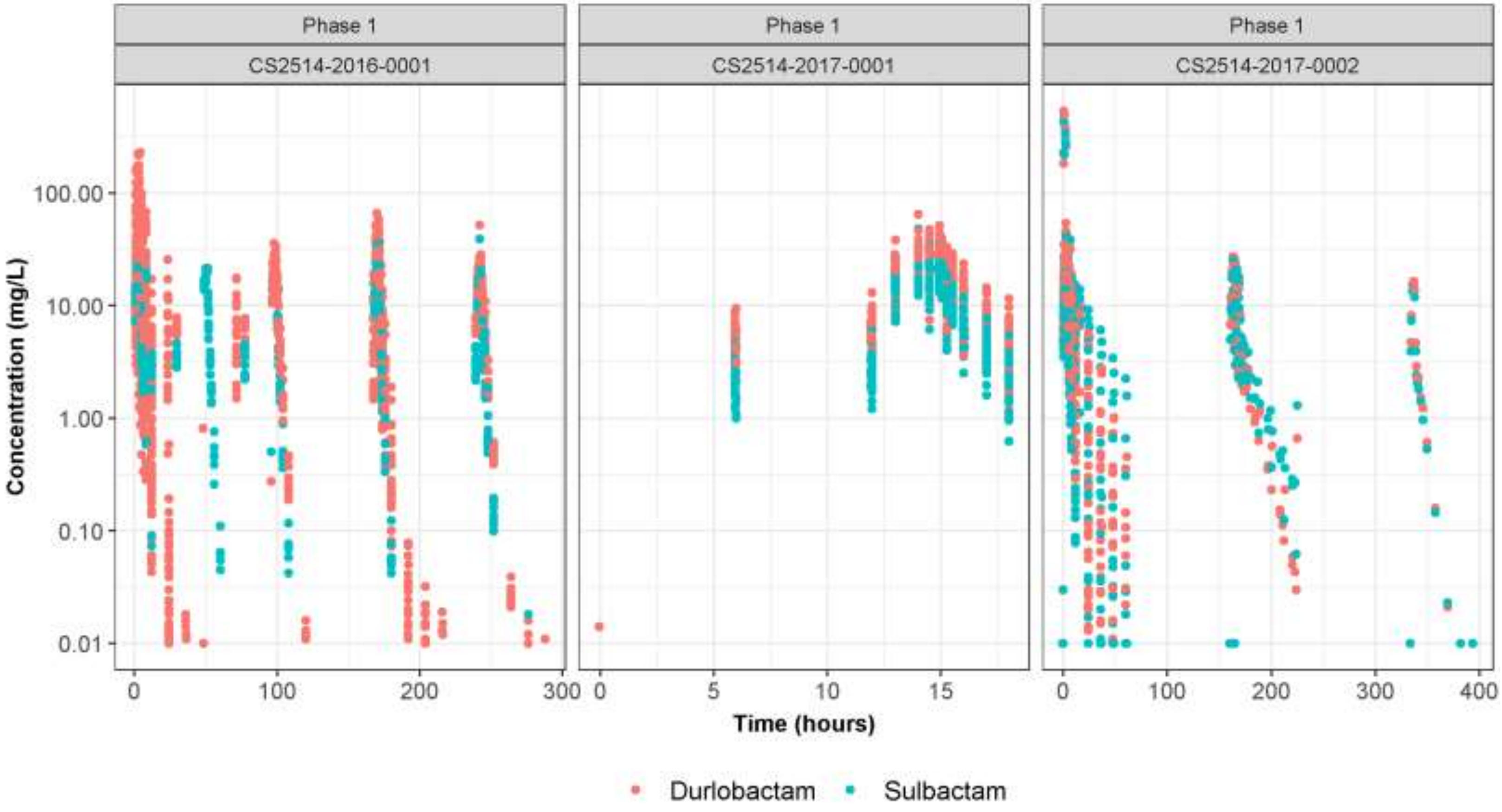

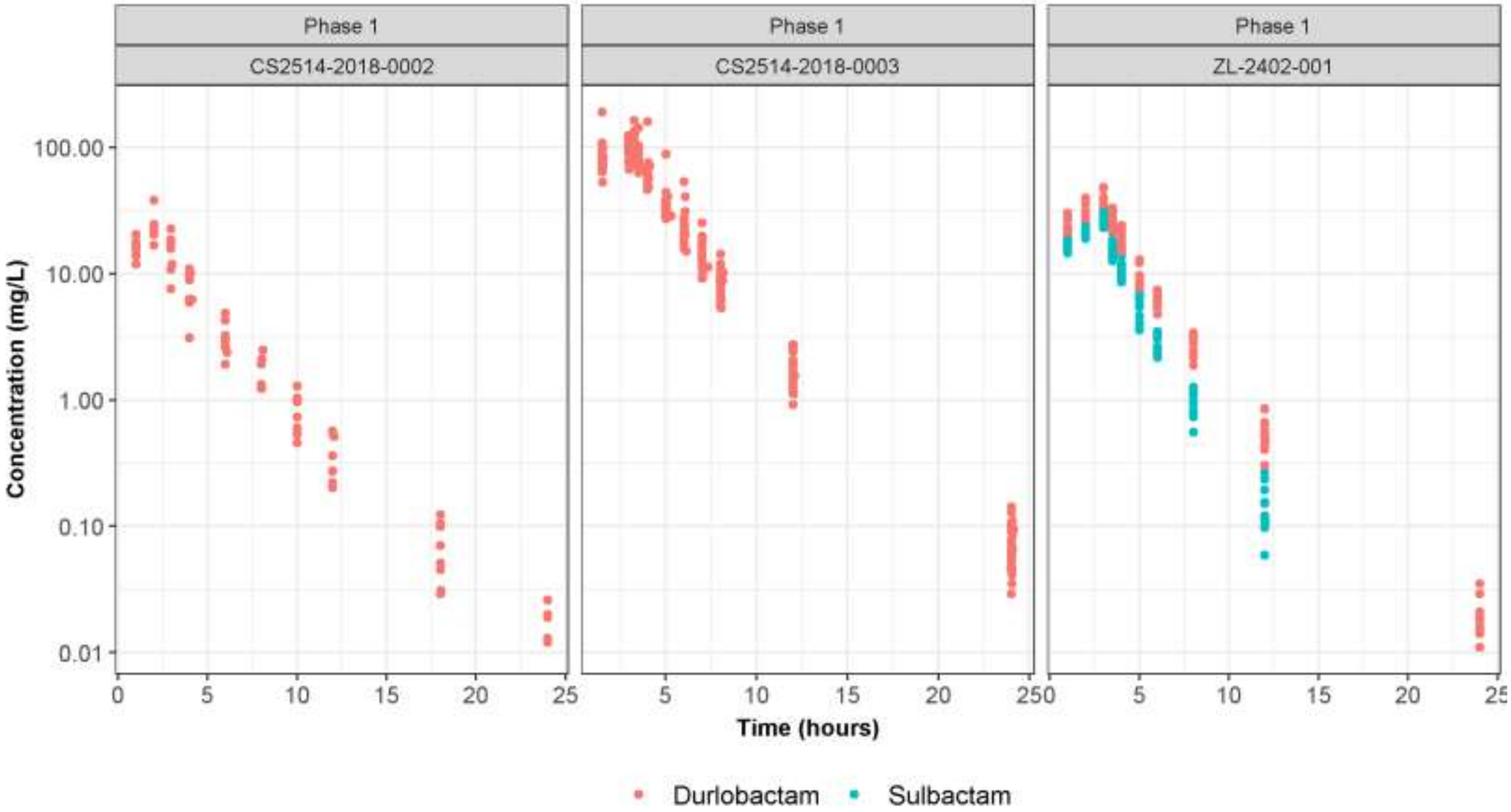
Phase 1
CS2514-2018-0002
Phase 1
CS2514-2018-0003
Phase 1
ZL-2402-001
Concentration (mg/L)
100.00
10.00
1.00
0.10
0.01
0
5
10
15
20
25
Time (hours)
Durlobactam
Sulbactam

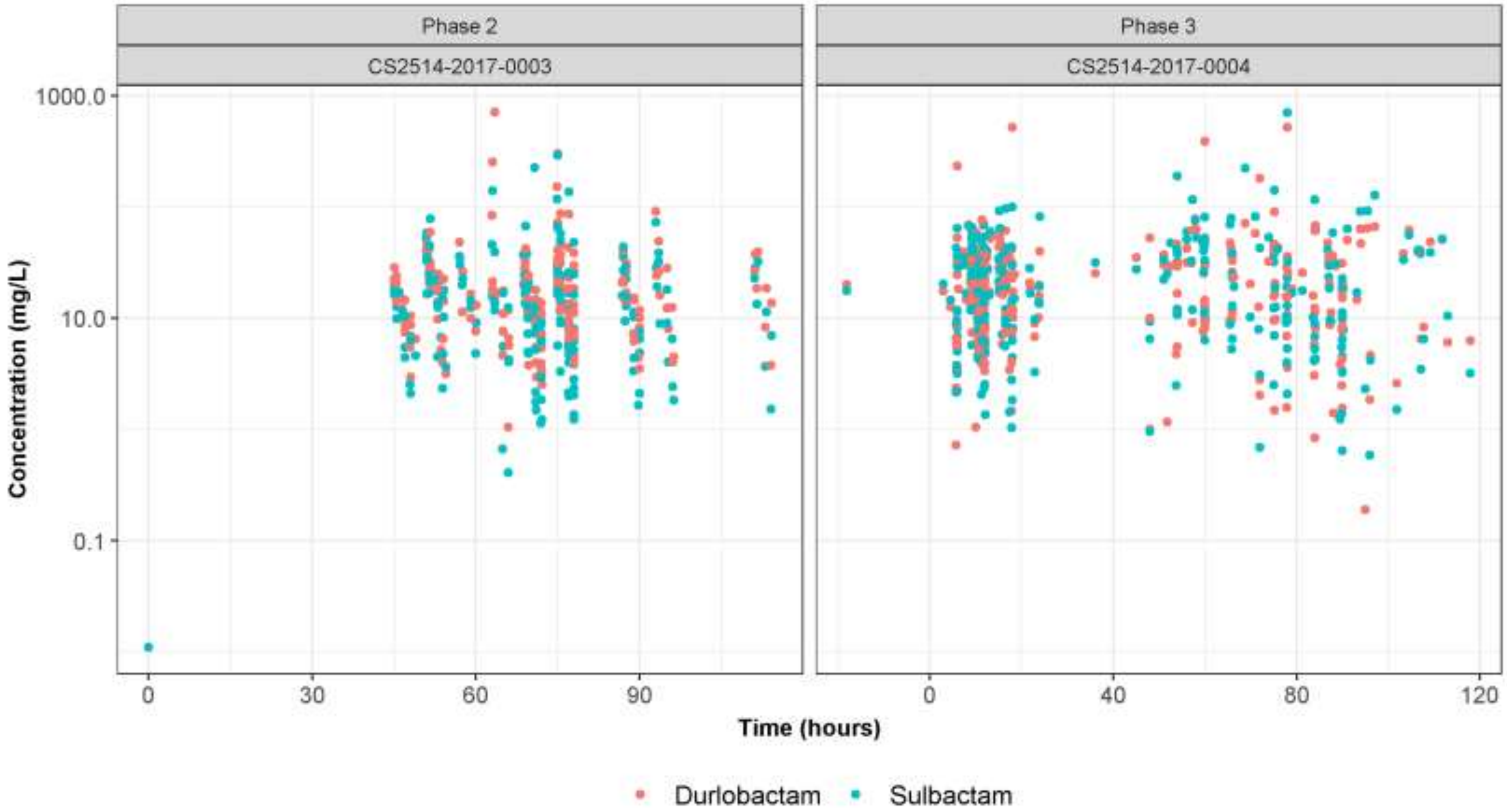

Note: Abbreviations are provided in the **Abbreviations Listing** on Pages 1-2.

Figure S3. Semi-log plot of plasma concentrations versus time since previous dose, stratified by Chinese study and paneled by analyte

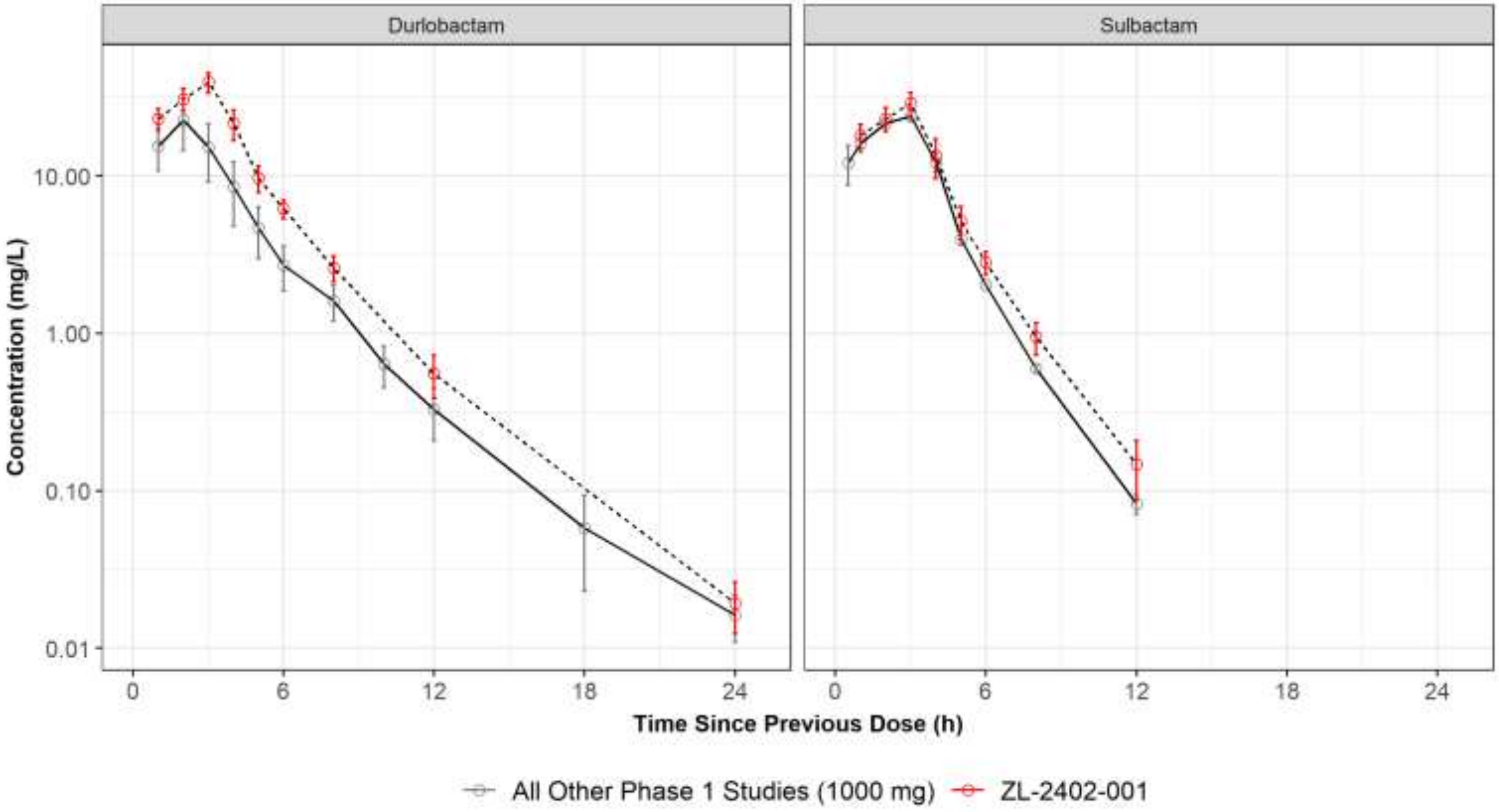

Note: Abbreviations are provided in the **Abbreviations Listing** on Pages 1-2. Open circles and error bars show mean ± SD at each nominal sampling time lines connect each point by group. Data for all other Phase 1 studies is limited to subjects with normal renal function who received 1/1 g doses of sulbactam-durlobactam.

Figure S4. Standard goodness-of-fit plots for the final population PK model (plasma concentrations) for durlobactam (Panel A) and sulbactam (Panel B)

A.

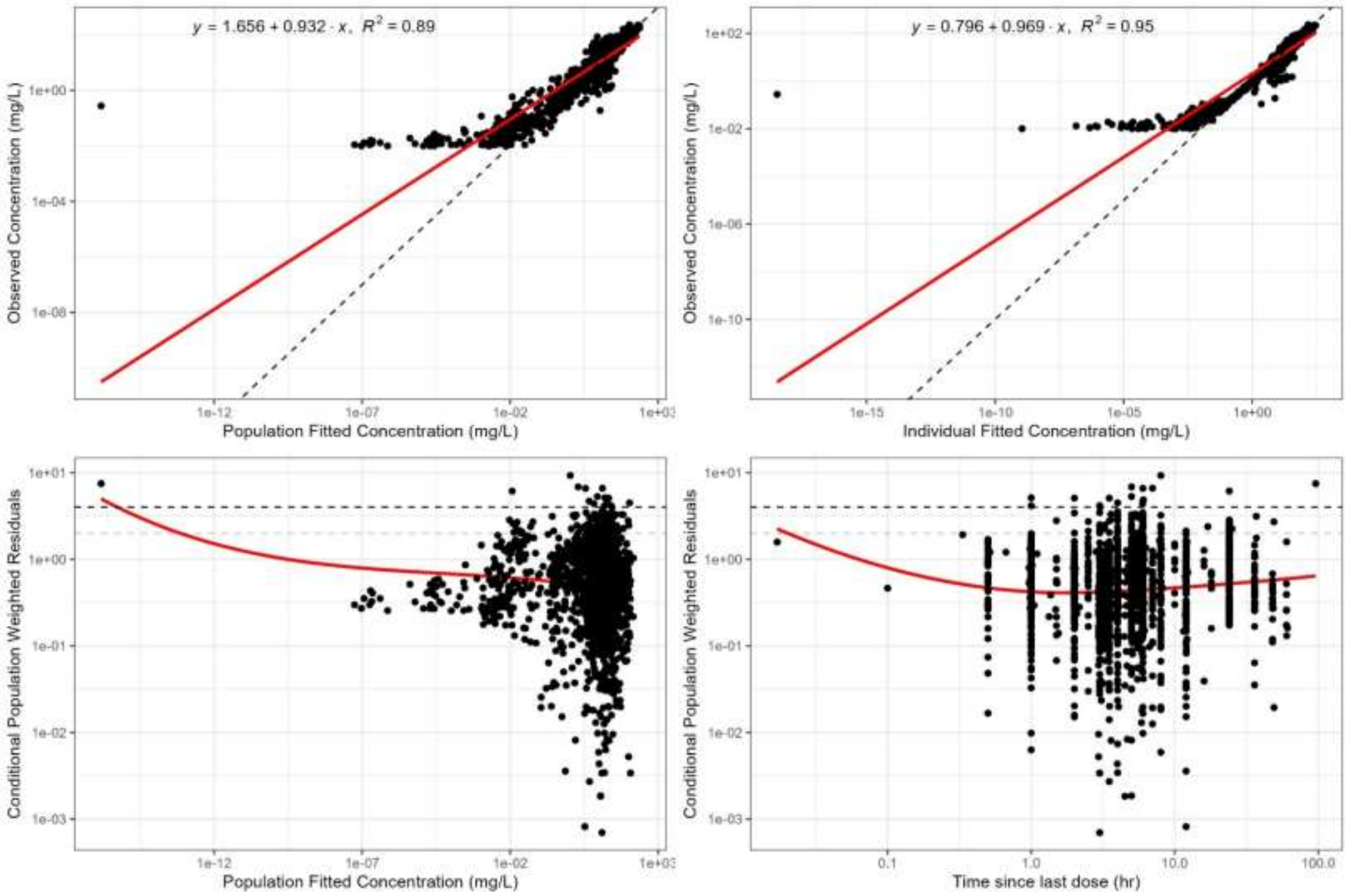

B.

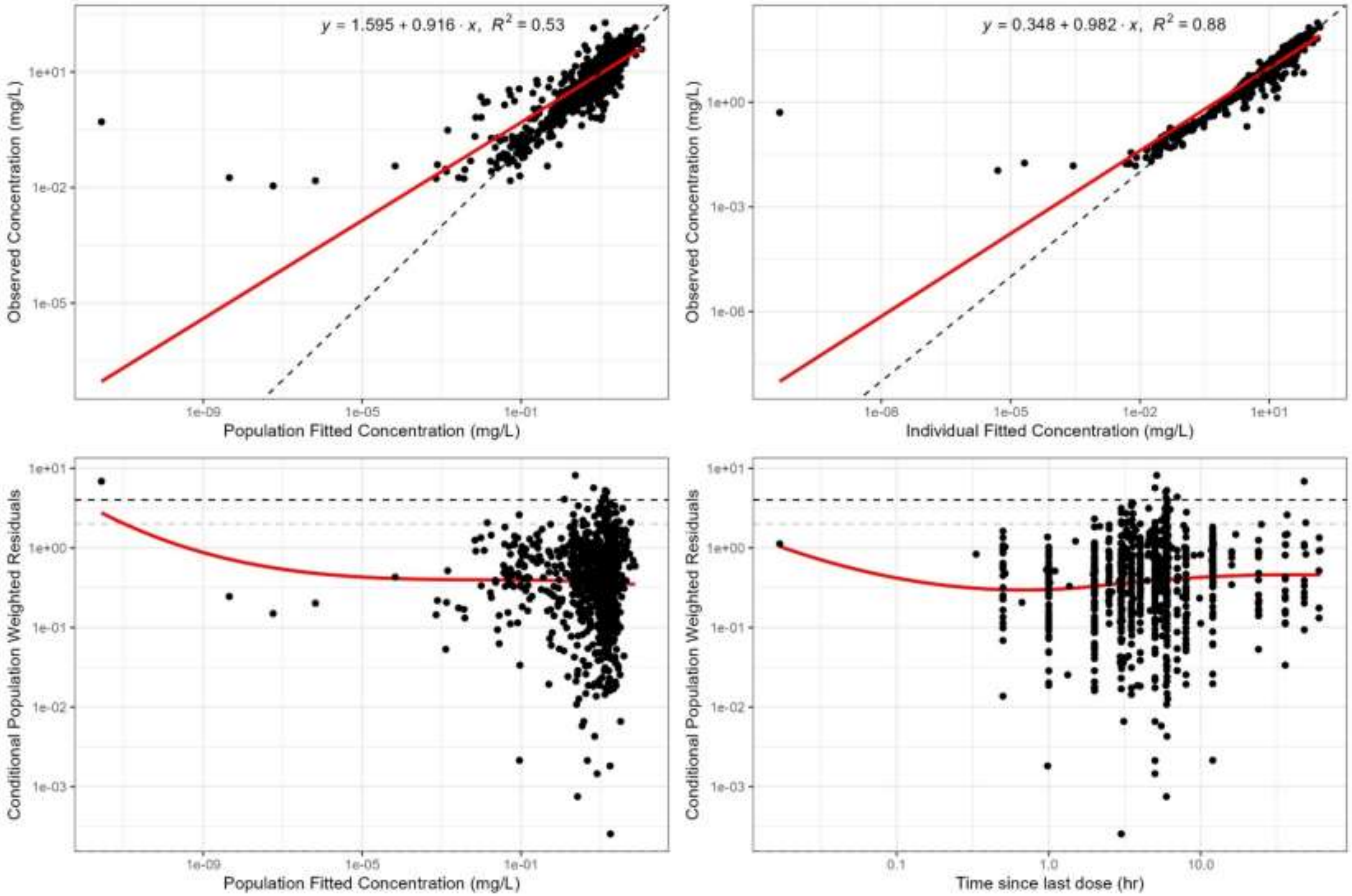

Note: Abbreviations are provided in the **Abbreviations Listing** on Pages 1-2.

Figure S5. Histograms of NPDE by dose (top) and scatterplot of NPDE by time (bottom) for the final population PK model fit to the pooled Phase 1, 2, and 3 data

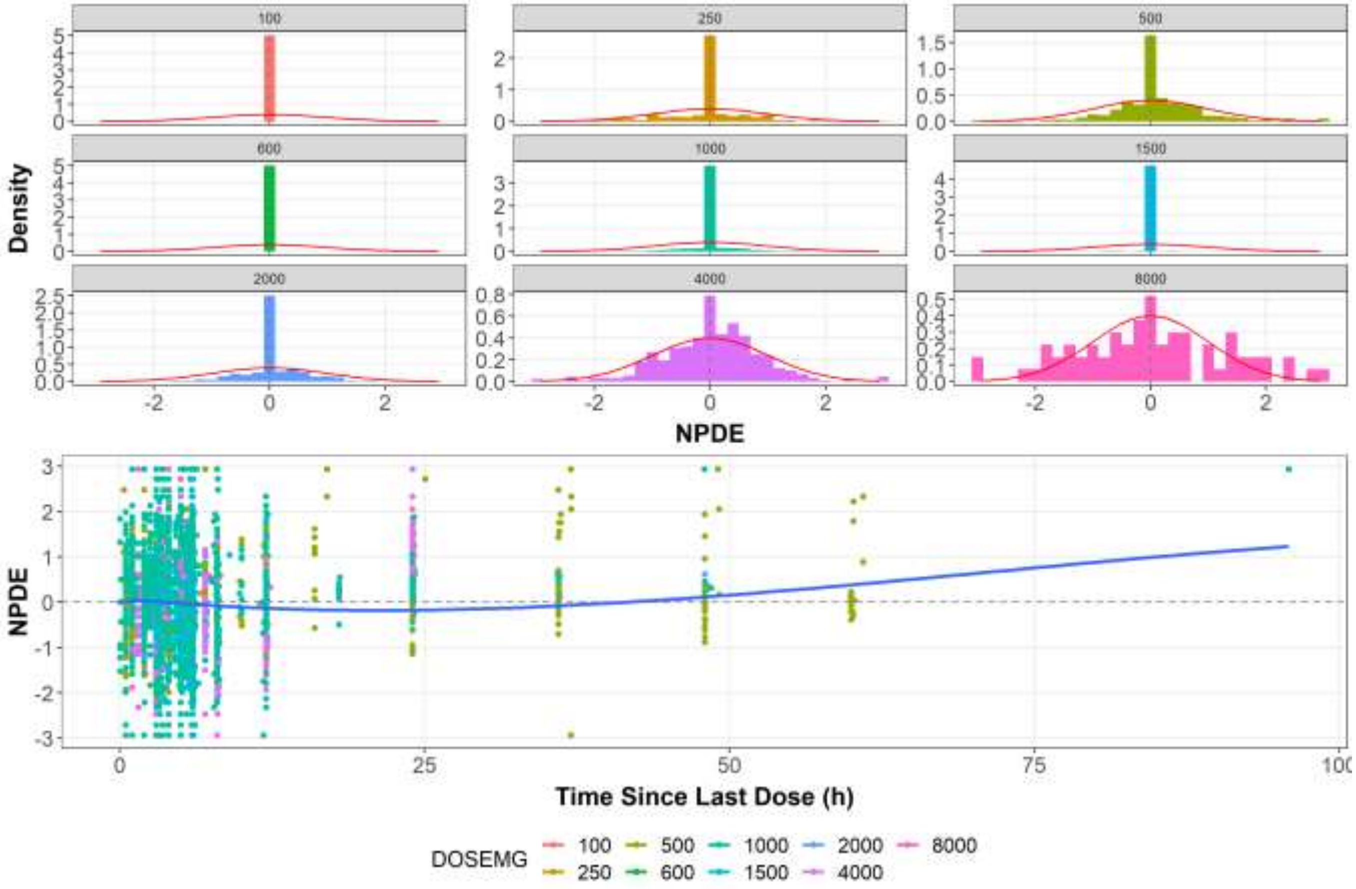

Note: Abbreviations are provided in the **Abbreviations Listing** on Pages 1-2.

Figure S6. Prediction-corrected visual predictive check plot for the final model of durlobactam (A) and sulbactam (B) using the pooled analysis dataset of healthy subjects

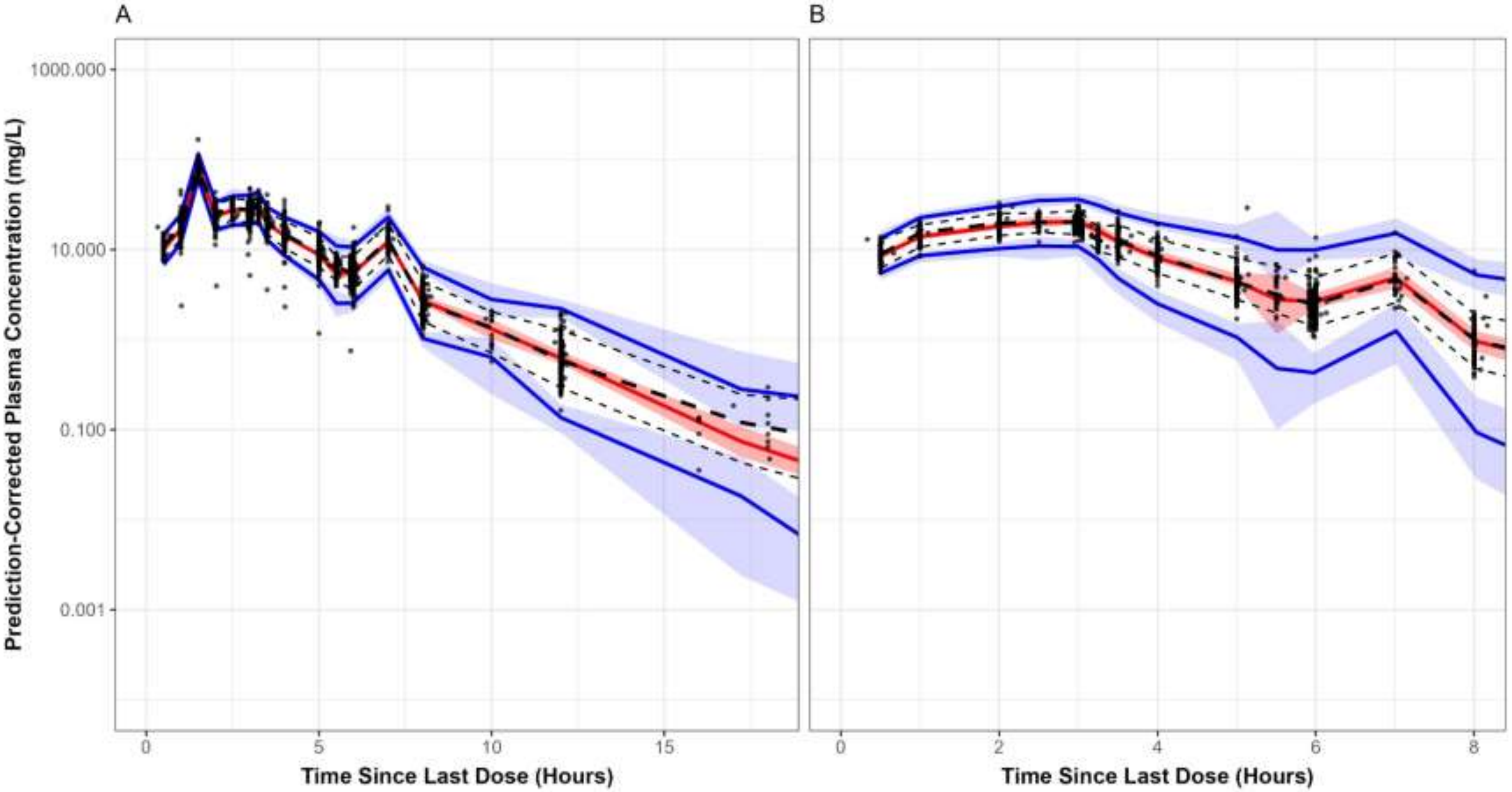

Note: Abbreviations are provided in the **Abbreviations Listing**. Circles are observed concentrations, black long dashed lines are the median observed concentrations, black short dashed lines are the 5th and 95th percentiles of the observed concentrations. Red and blue shaded regions are the 90% confidence intervals for the median, 5th, and 95th percentiles from the simulations on Pages 1-2; the red and blue lines represent the medians of these regions.

Figure S7. Prediction-corrected visual predictive check plot for the final model of durlobactam (A) and sulbactam (B) using the pooled analysis dataset of infected patients

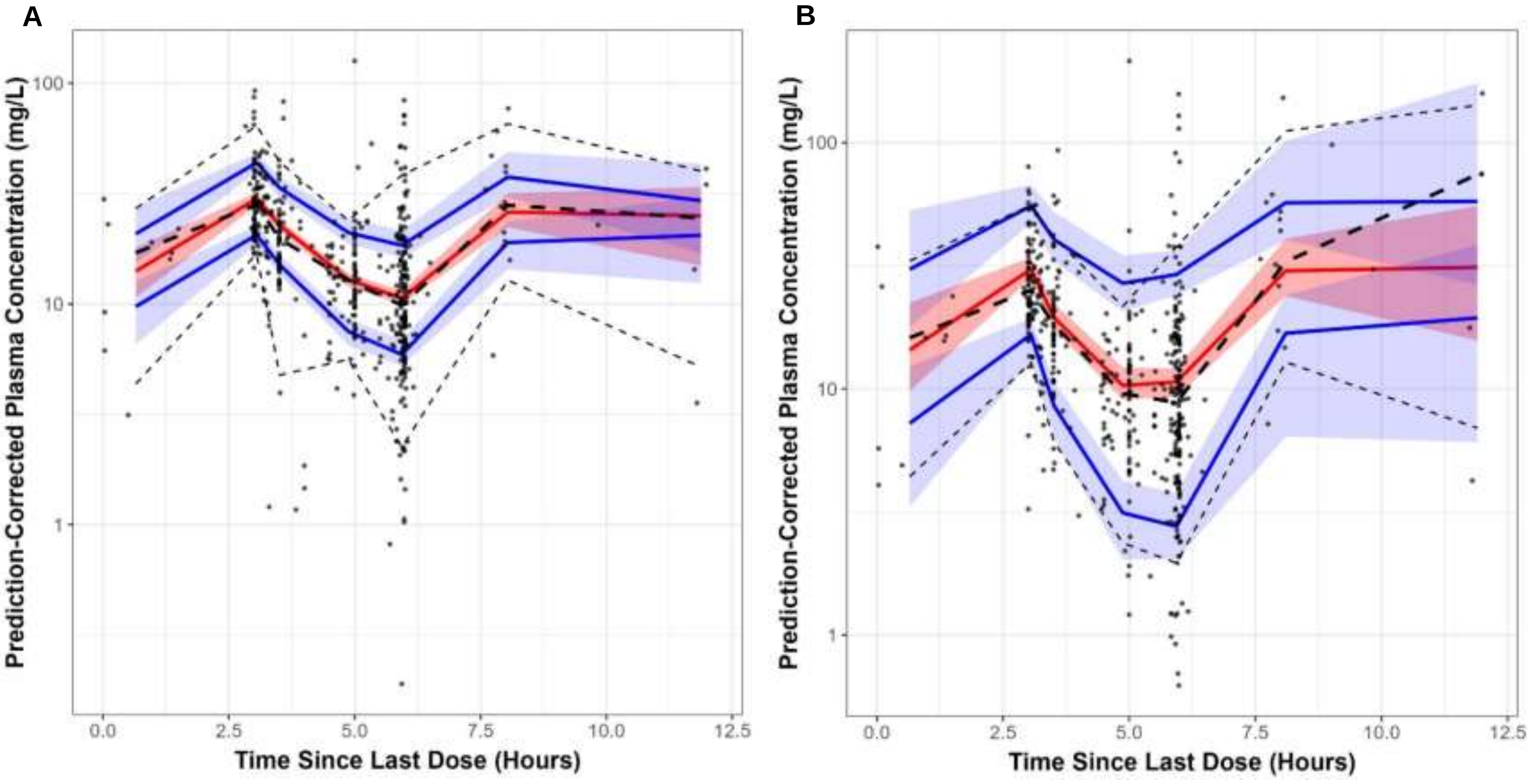

Note: Abbreviations are provided in the **Abbreviations Listing** on Pages 1-2. Circles are observed concentrations, black long dashed lines are the median observed concentrations, black short dashed lines are the 5th and 95th percentiles of the observed concentrations. Red and blue shaded regions are the 90% confidence intervals for the median, 5th, and 95th percentiles from the simulations; the red and blue lines represent the medians of these regions.

Figure S8. Standard goodness-of-fit plots for the durlobactam hemodialysis sub-model

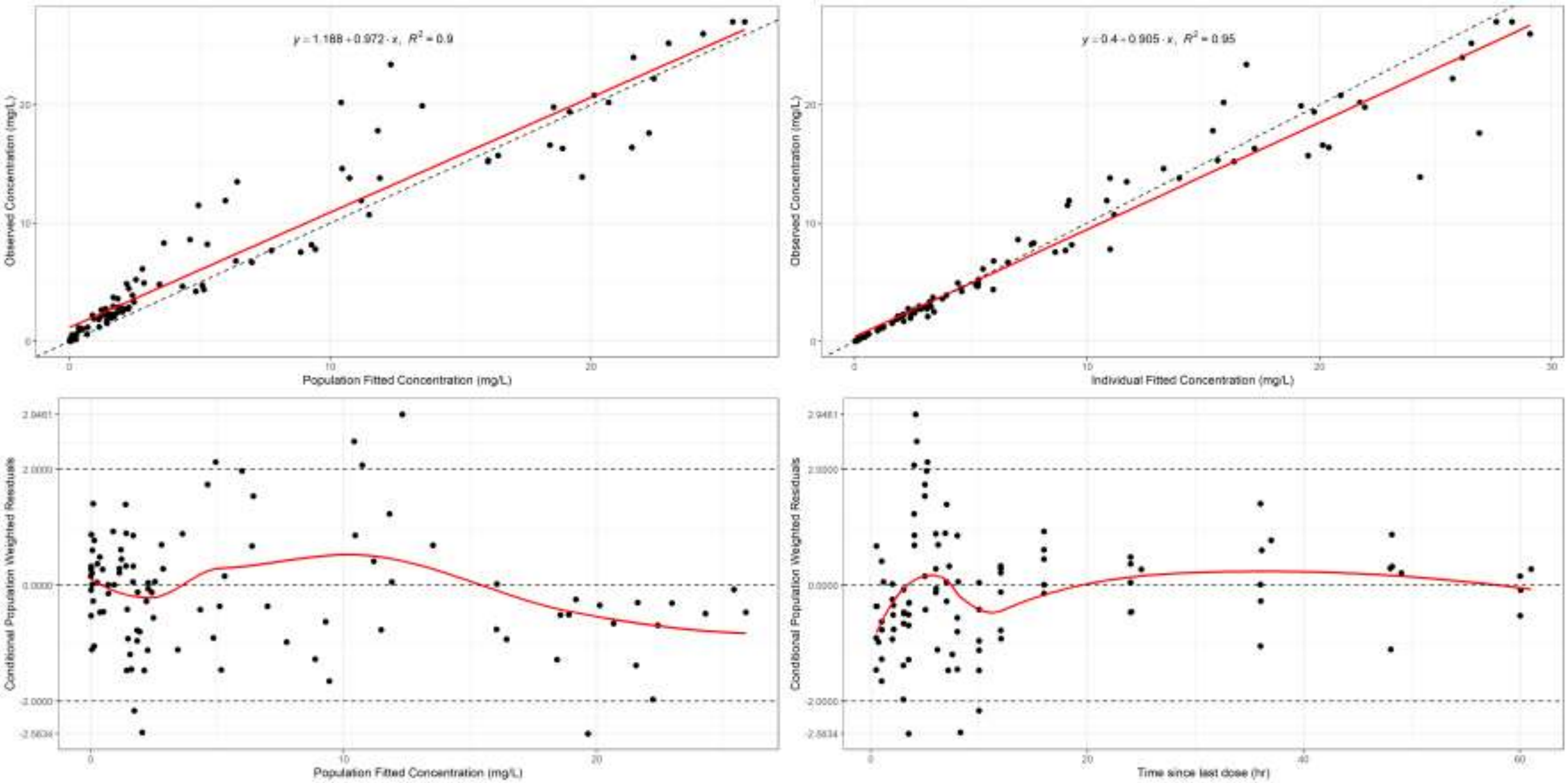

Note: Abbreviations are provided in the **Abbreviations Listing** on Pages 1-2.

Figure S9. Standard goodness-of-fit plots for the sulbactam hemodialysis sub-model

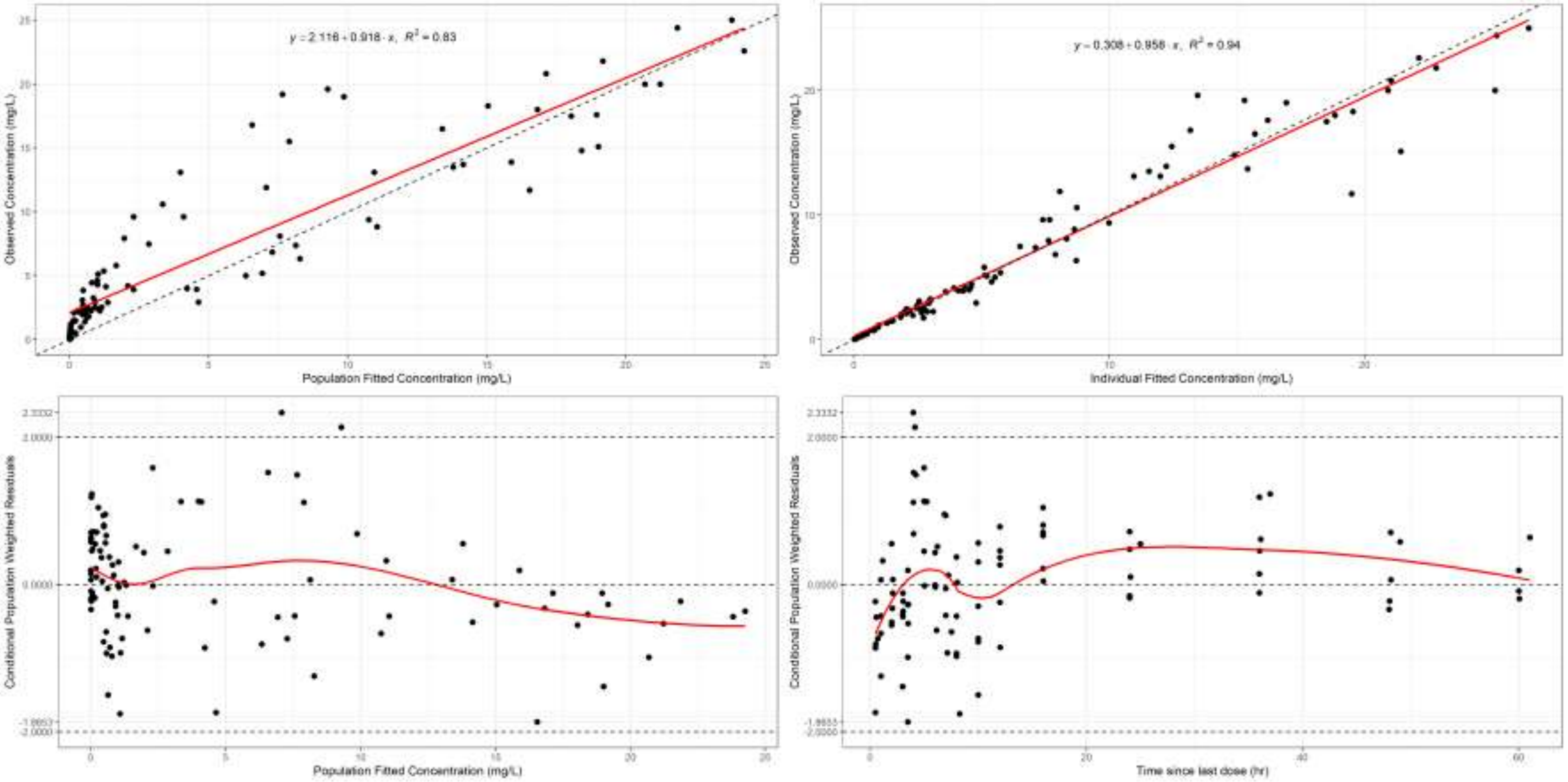

Note: Abbreviations are provided in the **Abbreviations Listing** on Pages 1-2.

Figure S10. Standard goodness-of-fit plots for the durlobactam ELF sub-model

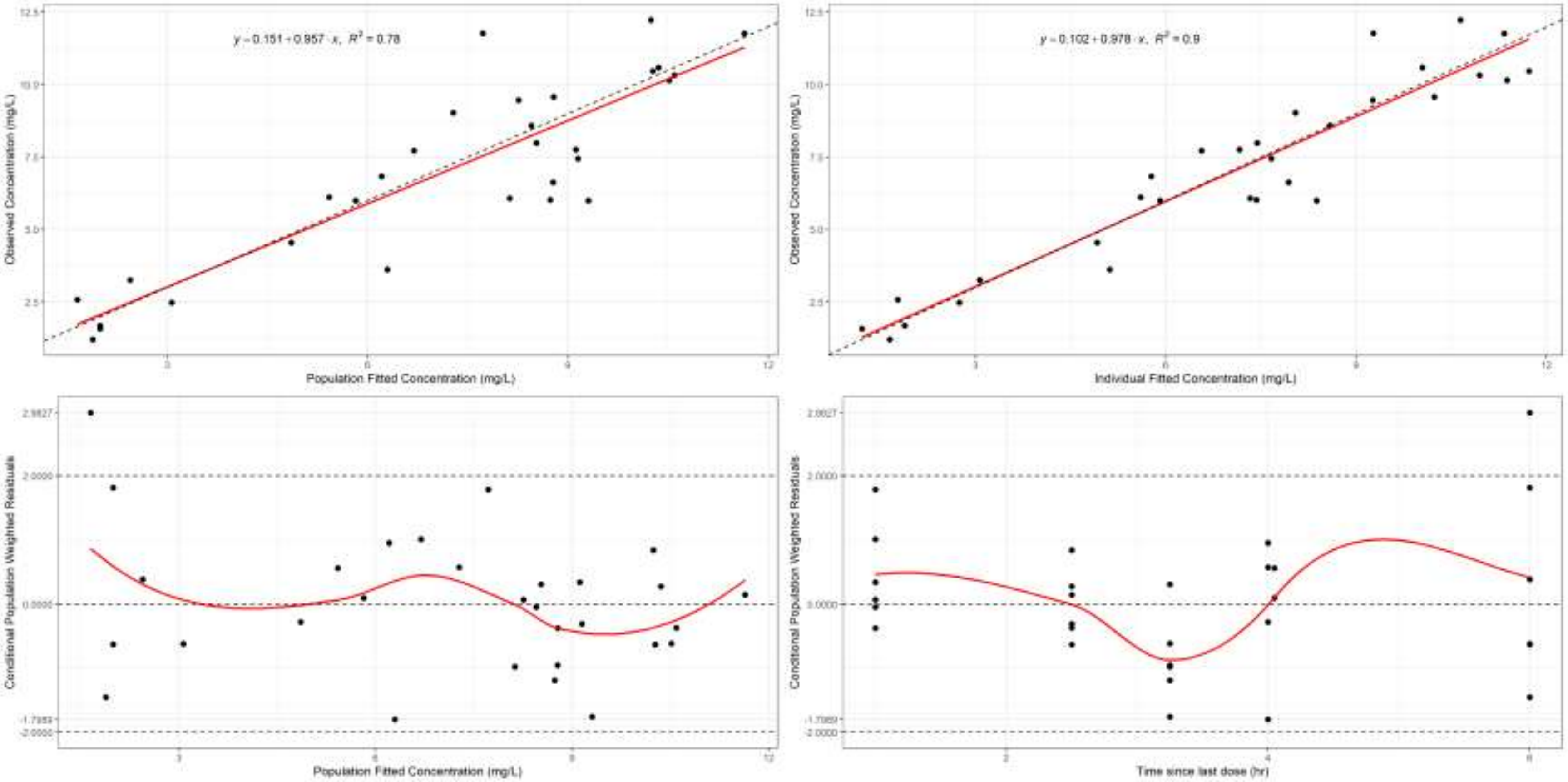

Note: Abbreviations are provided in the **Abbreviations Listing** on Pages 1-2.

Figure S11. Standard goodness-of-fit plots for the sulbactam ELF penetration sub-model

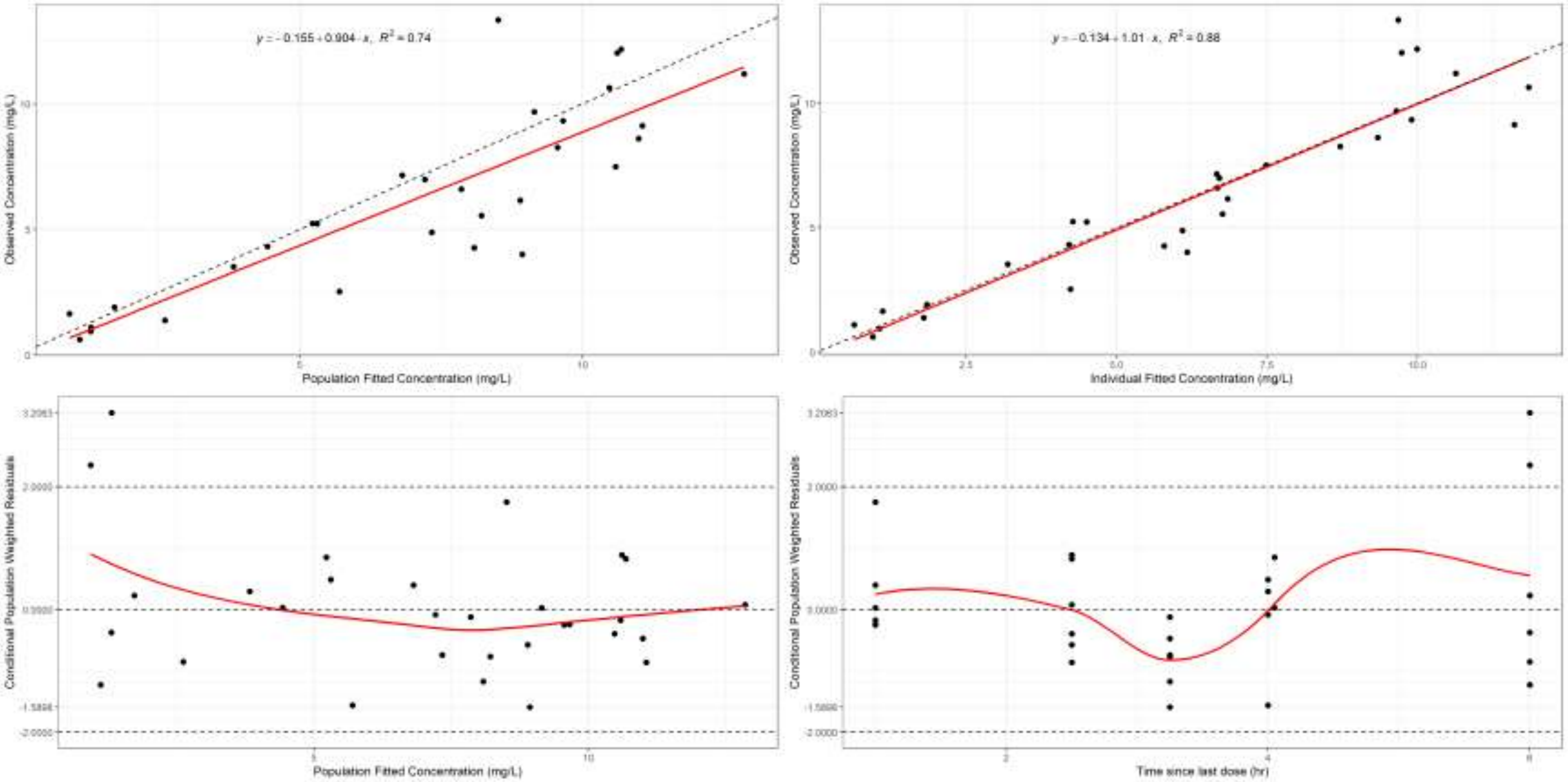

Note: Abbreviations are provided in the **Abbreviations Listing** on Pages 1-2.

Figure S12. Box-and-whisker plots showing the distributions of AUC_{0-24} and C_{max} by day and renal function category

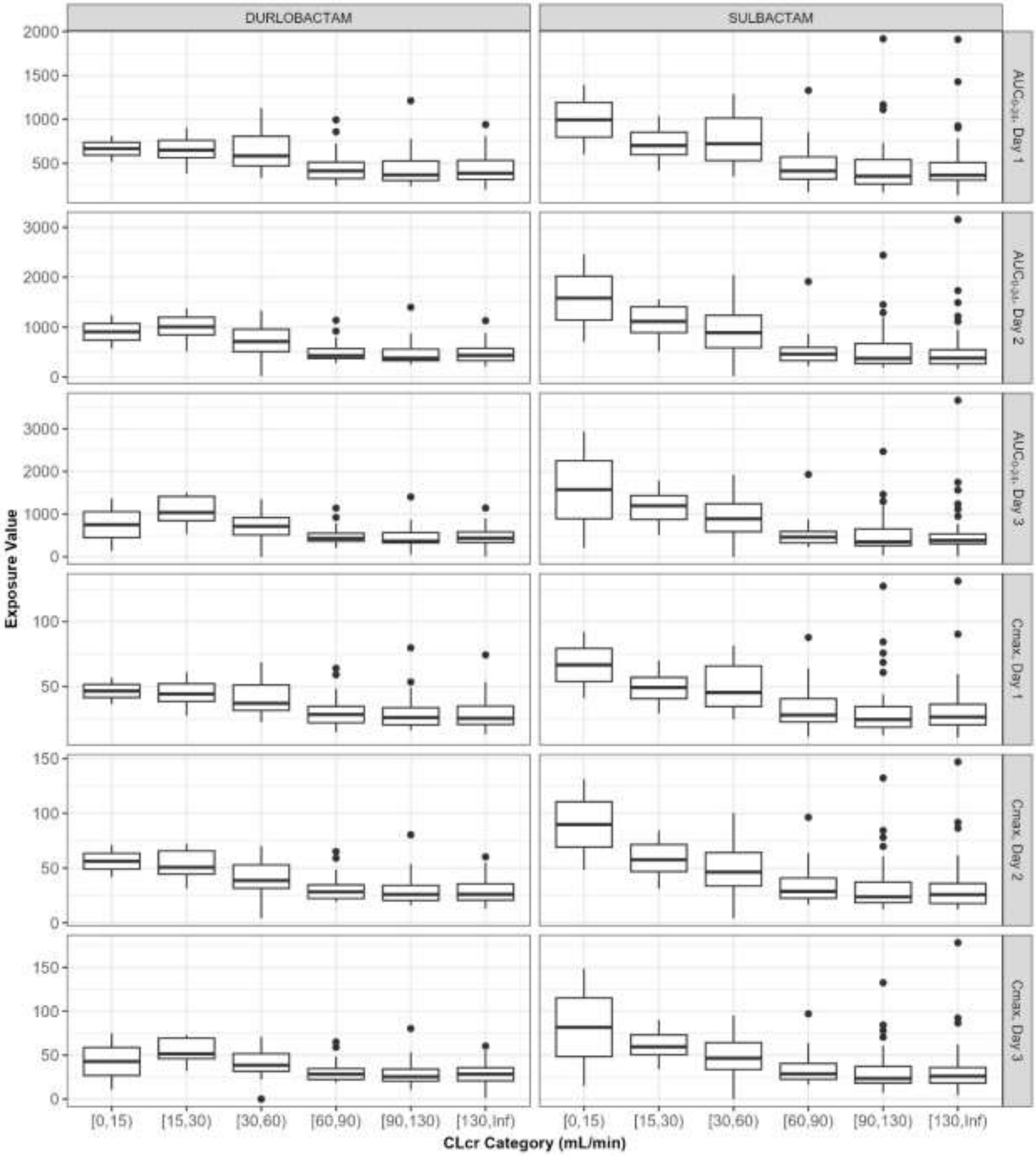

Note: Abbreviations are provided in the **Abbreviations Listing** on Pages 1-2. Counts of observations per group are provided in Table 3 in the manuscript.

Figure S13. Relationship between $AUC_{0\text{-}24}$ on Day 1 and body weight in patients

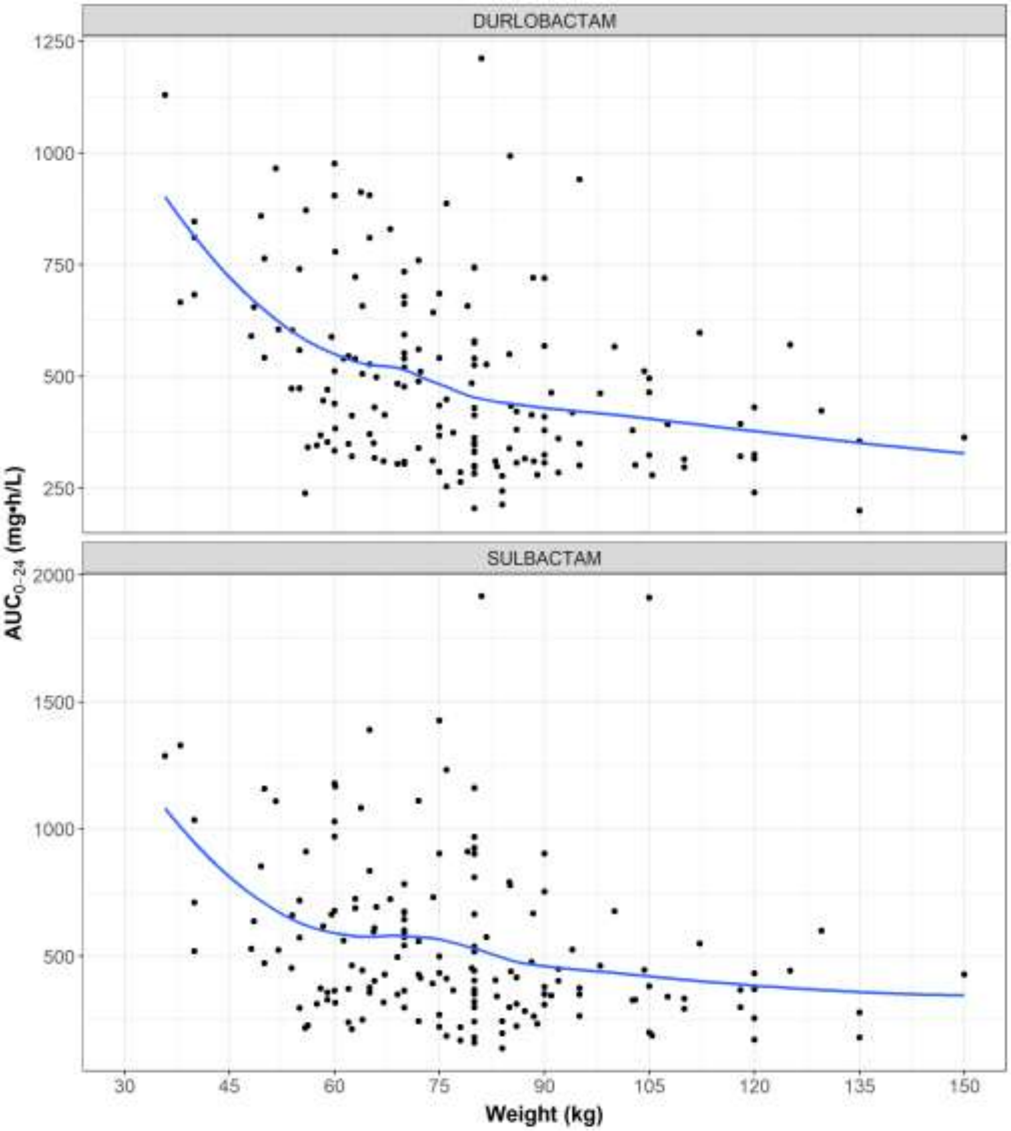

Note: Abbreviations are provided in the **Abbreviations Listing** on Pages 1-2.

Figure S14. Relationship between AUC_{0-24} on Day 1 and BMI in infected subjects.

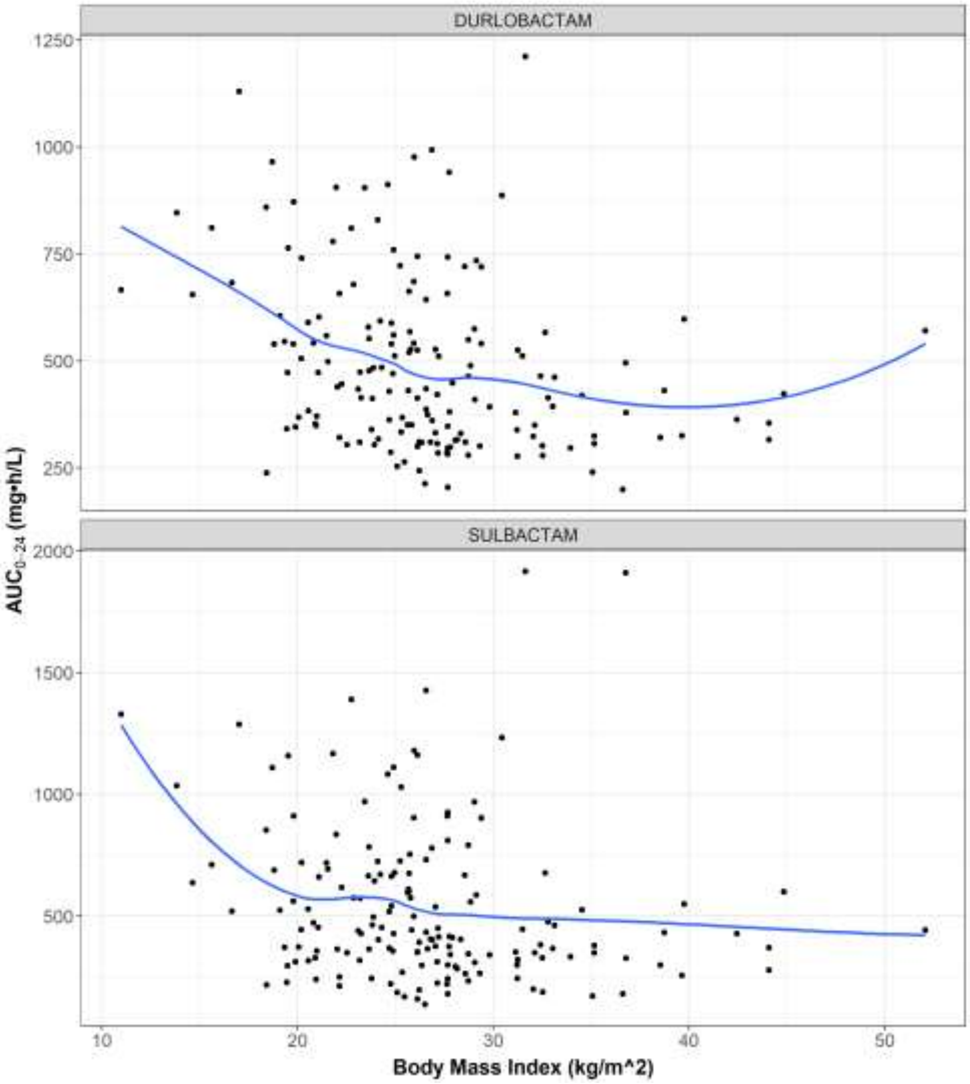

Note: Abbreviations are provided in the **Abbreviations Listing** on Pages 1-2.

Figure S15. Relationship between AUC_{0-24} on Day 1 and age in infected subjects

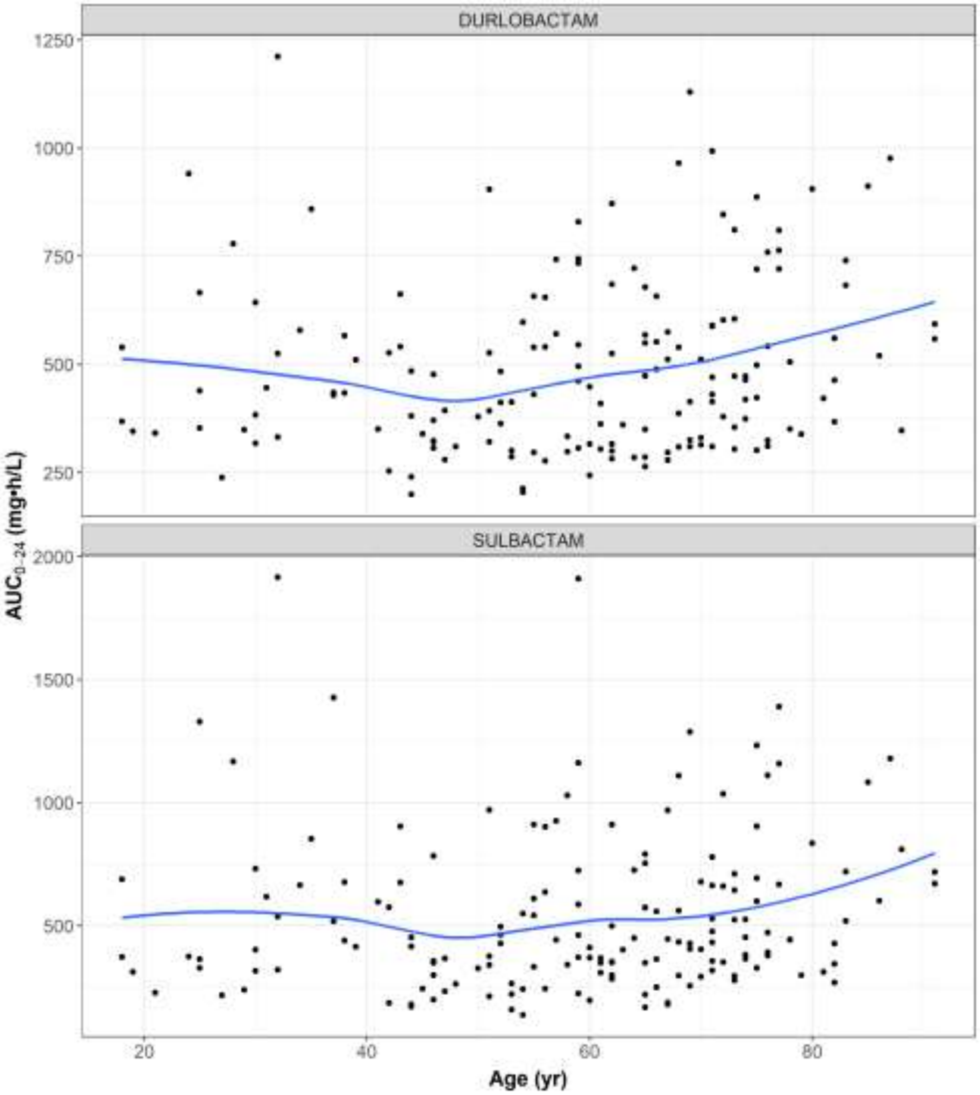

Note: Abbreviations are provided in the **Abbreviations Listing** on Pages 1-2.

Figure S16. Box-and-whisker plots showing the distributions of AUC_{0-24} and C_{max} by day and infection type

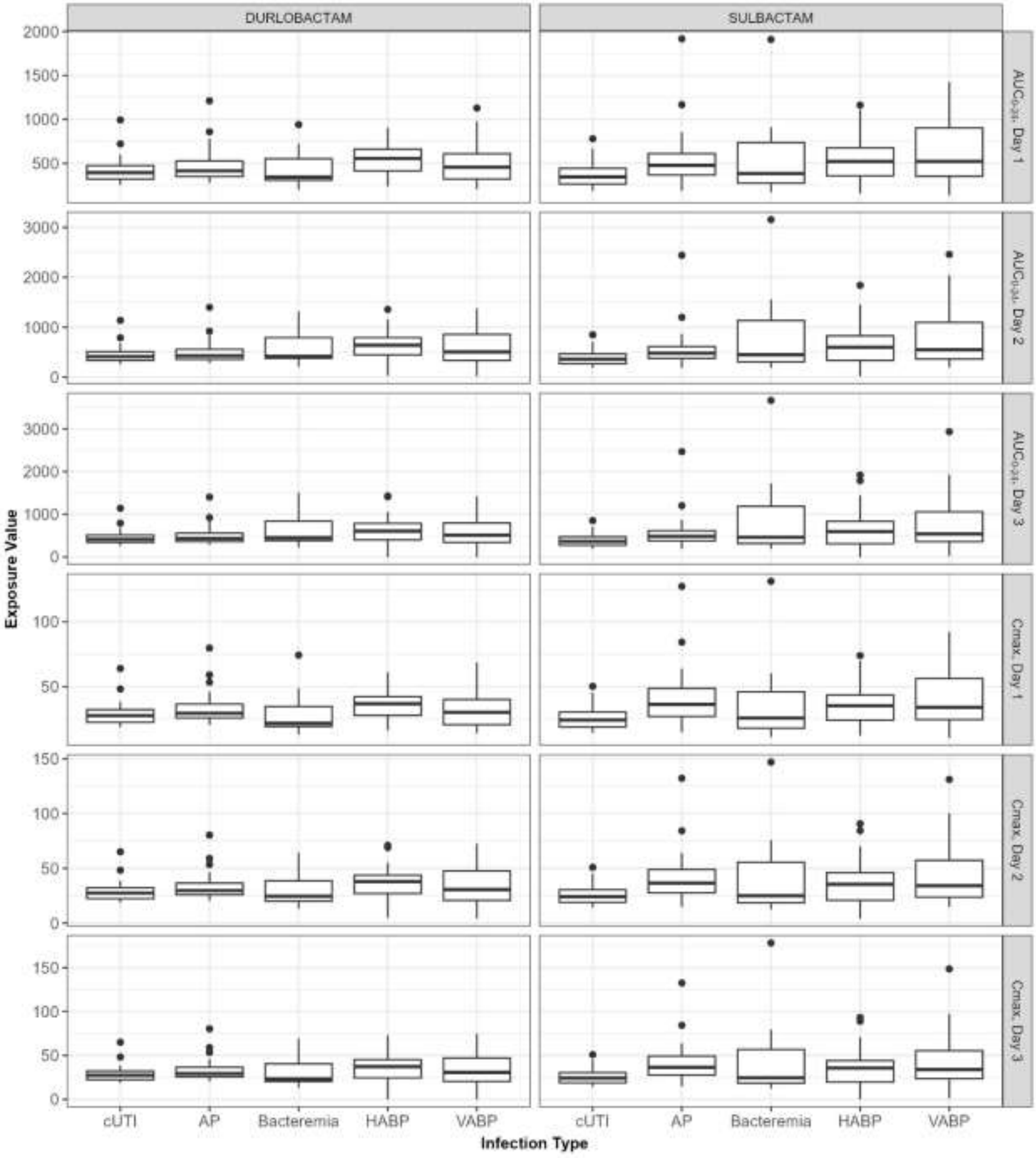

Note: Abbreviations are provided in the **Abbreviations Listing** on Pages 1-2. Counts of observations per group are provided in Table 5 in the manuscript.

Figure S17. Box-and-whisker plots showing the distributions of $AUC_{0\text{-}24}$ and C_{max} by day, region, and study phase

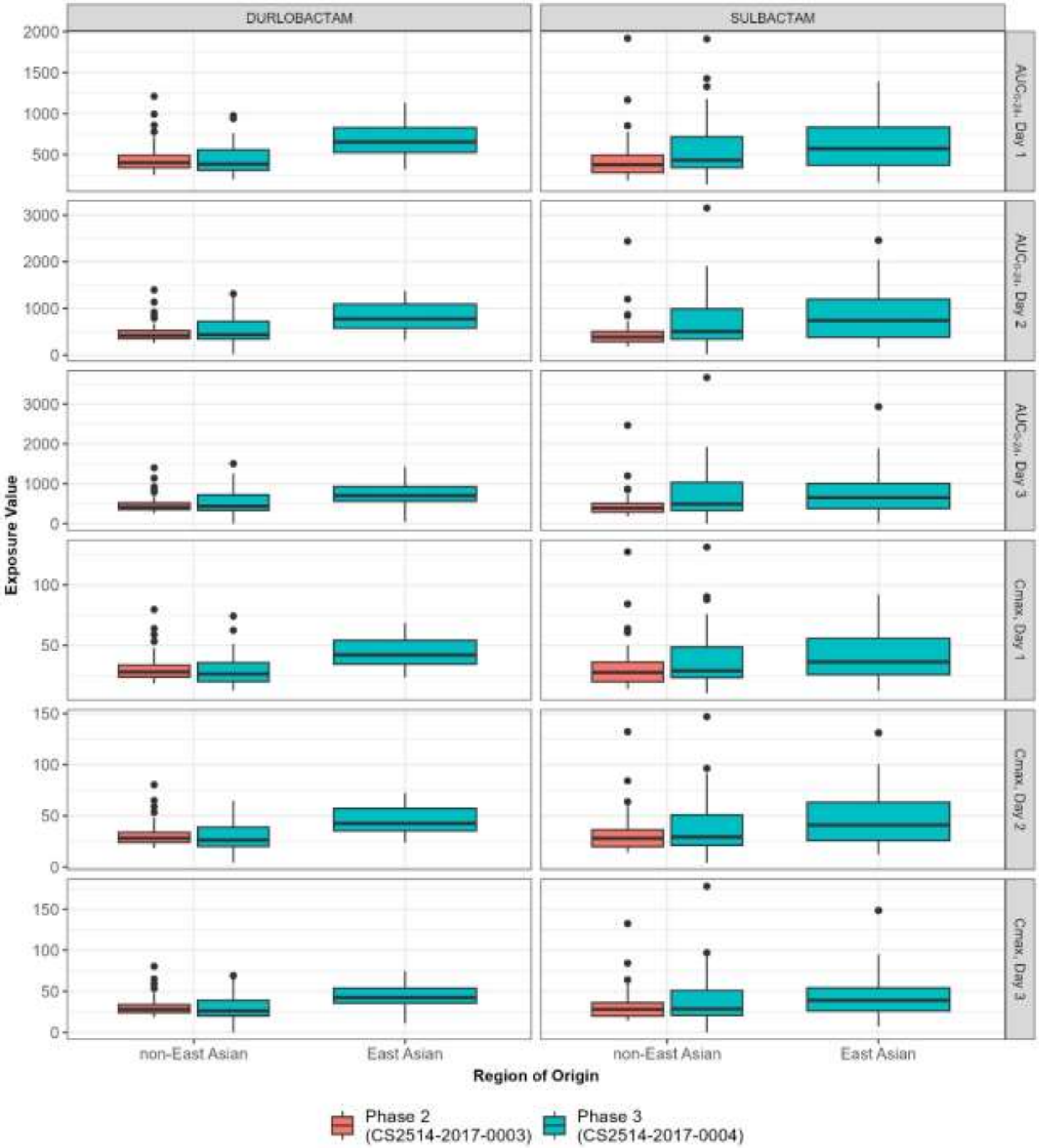

Note: Abbreviations are provided in the **Abbreviations Listing** on Pages 1-2. Counts of observations per group are provided in Table S10.

Figure S18. Schematics of both durlobactam and sulbactam base structural models applied to the pooled Phase 1, 2, and 3 data

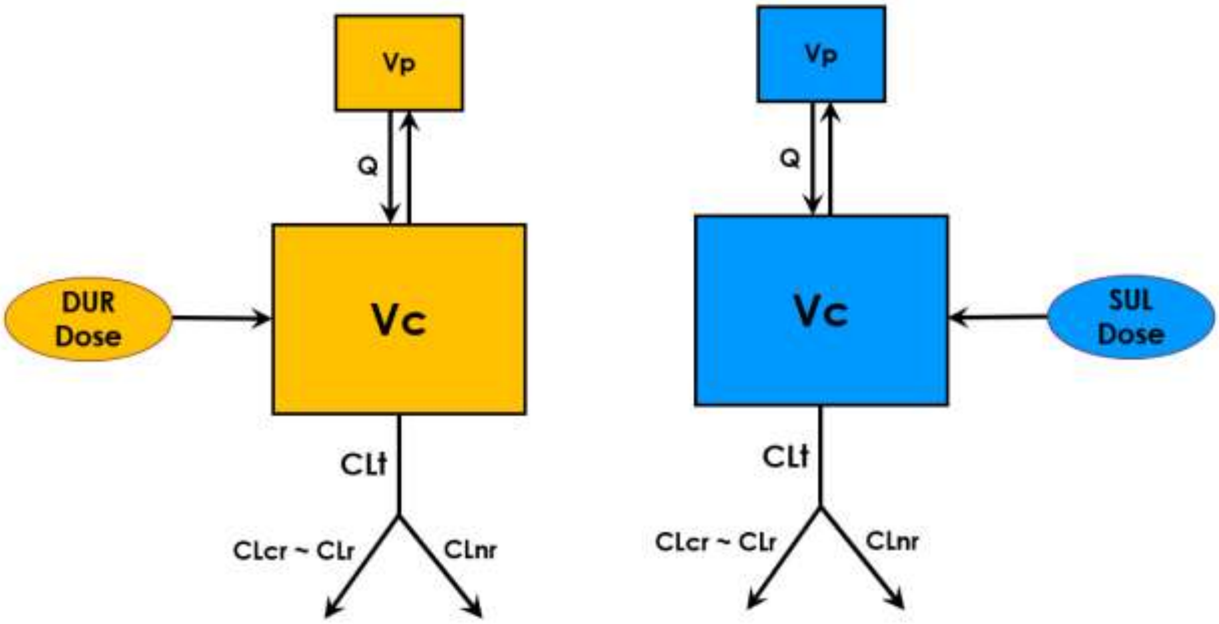

Note: Abbreviations are provided in the **Abbreviations Listing** on Pages 1-2.

Figure S19. dOFV distribution plot

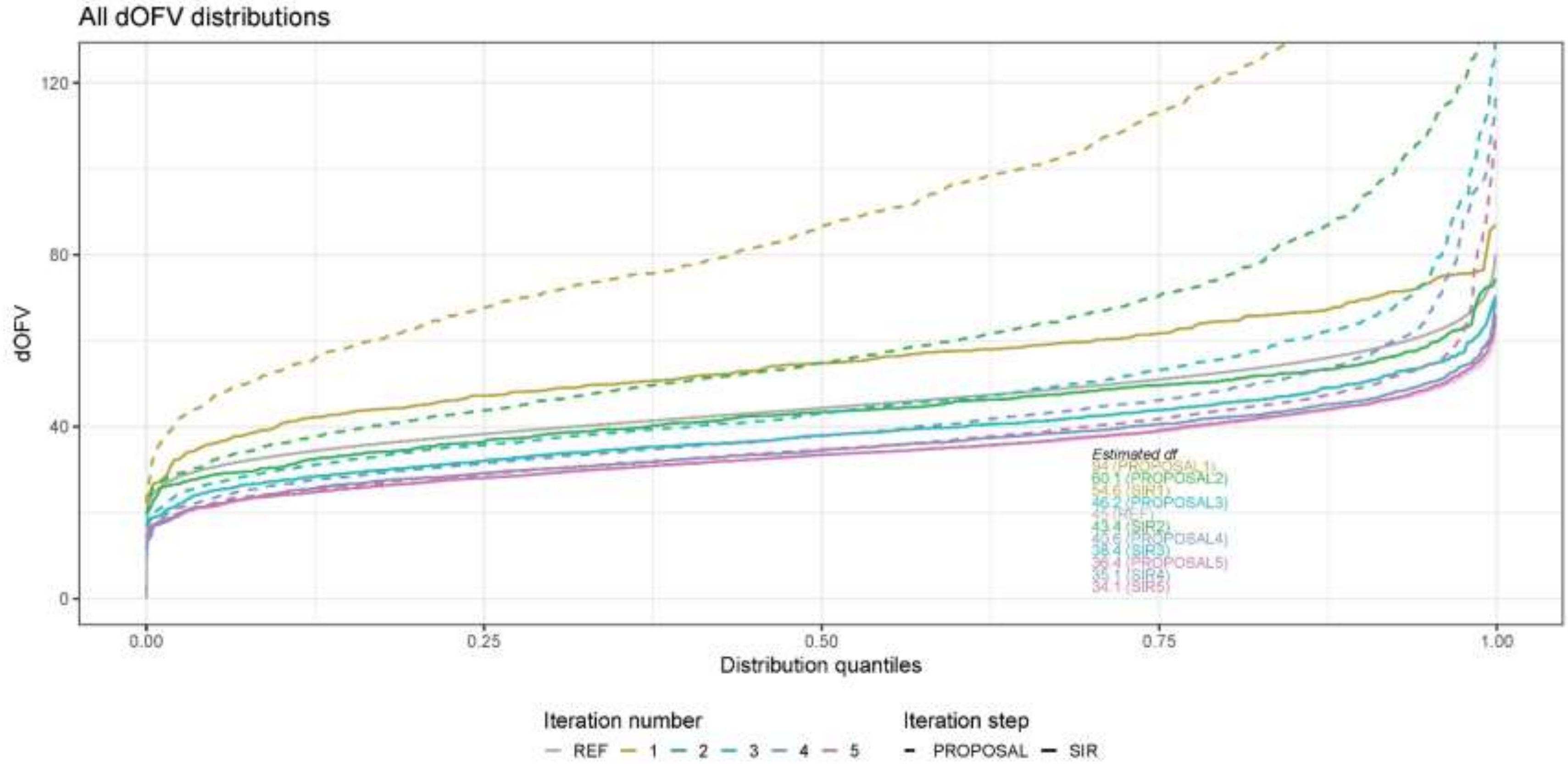

Supplement: Supplemental material — Tables S1 to S13 and Figures S1 to S19. [file aac.00485-24-s0001.pdf]
